# Influenza Virus Affects Intestinal Microbiota and Secondary *Salmonella* Infection in the Gut through Type I Interferons

**DOI:** 10.1371/journal.ppat.1005572

**Published:** 2016-05-05

**Authors:** Elisa Deriu, Gayle M. Boxx, Xuesong He, Calvin Pan, Sammy David Benavidez, Lujia Cen, Nora Rozengurt, Wenyuan Shi, Genhong Cheng

**Affiliations:** 1 Department of Microbiology, Immunology and Molecular Genetics, University of California, Los Angeles, Los Angeles, California, United States of America; 2 School of Dentistry, University of California, Los Angeles, Los Angeles, California, United States of America; 3 Department of Human Genetics, University of California, Los Angeles, Los Angeles, California, United States of America; 4 Department of Pathology and Laboratory Medicine, CURE Imaging and Stem Cell Biology Core, University of California, Los Angeles, Los Angeles, California, United States of America; University of California, Davis, UNITED STATES

## Abstract

Human influenza viruses replicate almost exclusively in the respiratory tract, yet infected individuals may also develop gastrointestinal symptoms, such as vomiting and diarrhea. However, the molecular mechanisms remain incompletely defined. Using an influenza mouse model, we found that influenza pulmonary infection can significantly alter the intestinal microbiota profile through a mechanism dependent on type I interferons (IFN-Is). Notably, influenza-induced IFN-Is produced in the lungs promote the depletion of obligate anaerobic bacteria and the enrichment of Proteobacteria in the gut, leading to a “dysbiotic” microenvironment. Additionally, we provide evidence that IFN-Is induced in the lungs during influenza pulmonary infection inhibit the antimicrobial and inflammatory responses in the gut during *Salmonella*-induced colitis, further enhancing *Salmonella* intestinal colonization and systemic dissemination. Thus, our studies demonstrate a systemic role for IFN-Is in regulating the host immune response in the gut during *Salmonella*-induced colitis and in altering the intestinal microbial balance after influenza infection.

## Author Summary

Influenza is a respiratory illness. Symptoms of flu include fever, headache, extreme tiredness, dry cough, sore throat, runny or stuffy nose, and muscle aches. Some people, especially children, can have additional gastrointestinal symptoms, such as nausea, vomiting, and diarrhea. In humans, there is no evidence that the influenza virus replicates in the intestine. Using an influenza mouse model, we found that influenza infection alters the intestinal microbial community through a mechanism dependent on type I interferons induced in the pulmonary tract. Futhermore, we demonstrate that influenza-induced type I interferons increase the host susceptibility to *Salmonella* intestinal colonization and dissemination during secondary *Salmonella*-induced colitis through suppression of host intestinal immunity.

## Introduction

Influenza is a highly contagious viral infection that has a substantial impact on global health.

Notably, outbreaks of influenza infection are usually associated with an increased incidence or severity of secondary bacterial infections responsible for high levels of morbidity during seasonal influenza episodes. We and others have previously shown that IFN-Is play a critical role in the development of secondary bacterial pneumonia after influenza infection [1]. Since their discovery in 1957, IFN-Is have been recognized as the central antiviral cytokines in vertebrates [2]. The type I IFN family mainly consists of numerous subtypes of IFNα and a single IFNβ, whose induction appears to be ubiquitous in most cell types. Toll-like receptor (TLR)-mediated IFN-I induction plays a key role in facilitating antiviral responses [3]. IFN-Is bind to a common heterodimeric receptor, IFN-α/β receptor (IFNAR), composed of two subunits, IFNAR1 and IFNAR2. Binding activates the JAK/STAT pathway, which induces pro-inflammatory genes that inhibit viral replication and boost adaptive immunity [4], and regulates the transcription of multiple interferon-stimulated genes (ISGs) [5].

A recent study has shown that influenza infection can alter the composition of the intestinal flora, resulting in immunological dysregulation that may promote inflammatory gut disorders [6]. The mammalian gut harbors a complex microbiota that plays a key role in host health through its contribution to nutritional, immunological, and physiological functions. Intestinal commensals are required for maintaining gut homeostasis through dynamic interactions with the host’s immune system [7]. Resident microbiota promote gut immune homeostasis by regulating T regulatory cells (Tregs) [8] and T helper 17 cells (Th17) [9]. The gut microbiota can inhibit infection through direct microbial antagonism or by stimulating several host effectors and injury responses [10, 11]. However, the intestinal commensals also pose an enormous challenge to the host that needs to remain “ignorant” to a selection of microbial antigens and keep the bacterial load anatomically contained, while remaining responsive to its dissemination [12]. Reciprocal interactions between gut microbiota and the host immune system shape the microbial community and influence imbalances that can lead to disease [13]. These changes are often characterized by a reduction of obligate anaerobic bacteria, and a proliferation of facultative anaerobic *Enterobacteriaceae* [14].

Furthermore, some people with pulmonary influenza infections also experience symptoms of gastrointestinal disorders, especially children [15]. Influenza RNA is rarely recovered from their stool [15], so it is unclear whether the symptoms develop from swallowed respiratory secretions or from active infection of the gastrointestinal tract.

In order to investigate the role of IFN-Is induced during influenza infection in modulating the endogenous intestinal microbiota, we established a model of influenza pulmonary infection using genetically modified animals with defective IFNAR signaling (*Ifnar1* ^–/–^mice). Remarkably, we found that influenza infection alters the intestinal microbial community supporting gut Proteobacteria pathobionts through a mechanism dependent on IFN-Is.

While the importance of IFN-Is in antiviral defense is well established, their role during bacterial infection is more ambiguous. Moreover, we wanted to test whether primary influenza infection can predispose the host to secondary intestinal bacterial infections. We therefore developed a model of sequential influenza pulmonary infection followed by secondary *Salmonella*-induced colitis using *Ifnar1* ^–/–^mice to investigate the effects of IFN-Is induced during influenza infection on intestinal host defense against *Salmonella*. Interestingly, we found that lung induced IFN-Is enhanced the growth of *Salmonella* in the inflamed gut and increased its systemic dissemination to secondary sites. Furthermore, we found that influenza pulmonary infection resulted in a profound inhibitory effect on the intestinal antibacterial and inflammatory responses against *Salmonella* infection in a IFN-I dependent manner.

## Results

### Influenza-induced IFN-Is alter the intestinal microbiota

Previously, it was shown that influenza infection causes intestinal injury through microbiota-dependent inflammation [6]. Considering that IFN-Is are essential components of the host antiviral response, we hypothesized that these molecules might also mediate changes in the intestinal microbiota during viral influenza infection. To study this, we infected wild-type (WT) and *Ifnar1* knockout (*Ifnar1* ^*-/-*^) mice by non-surgical intratracheal instillation [16, 17] with a sublethal dose (200 infectious units) of influenza A/Puerto Rico/8/34 (PR8).

**Fig 1:**
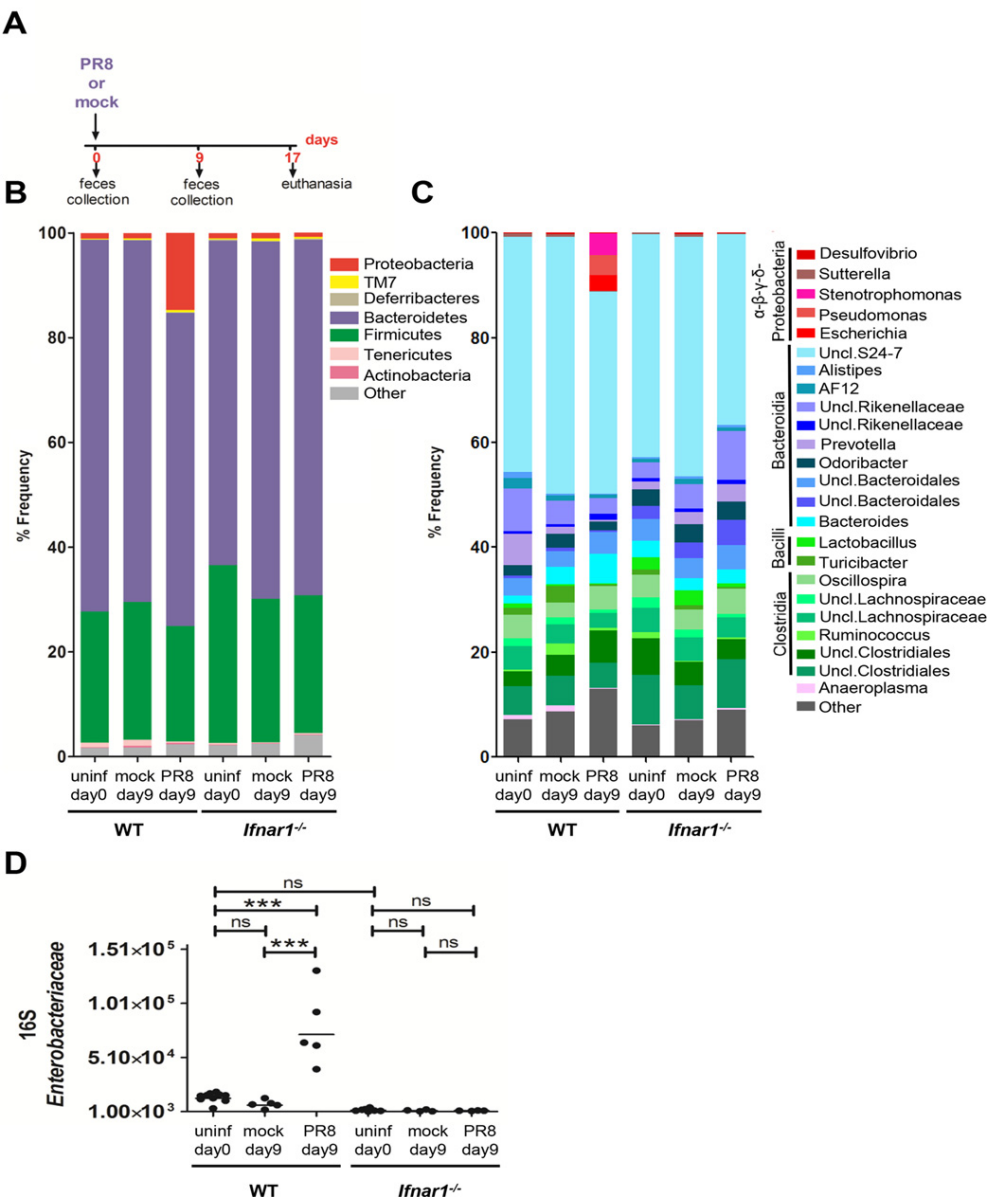
PR8-induced IFN-Is alter the fecal microbiota composition, Analysis of fecal microbiota in WT and *Ifnar1* ^*-/-*^ mice performed by MiSeq and 16S qPCR during influenza infection. A) Experimental model. Fecal samples were collected from mice on day 0 before infection and on day 9 after mock and PR8 infection. Mice were euthanized at 17 dpi. B, C) The fecal microbiota from WT and *Ifnar1* ^*-/-*^ mice on day 0 before infection (n = 6 WT, n = 6 *Ifnar1* ^*-/-*^), and on day 9 after mock (n = 3 WT, n = 3 *Ifnar1* ^*-/-*^) and PR8 infection (n = 3 WT, n = 3 *Ifnar1* ^*-/-*^) was analyzed by sequencing using the Illumina MiSeq system. Graphed is the average relative abundance of each bacterial phylum (B) and genus (C); the cut-off abundance level was set at 0.5%. D) Analysis of the fecal *Enterobacteriaceae* using 16S qPCR. Fecal samples were collected from mice on day 0 before infection (n = 10 WT, n = 8 *Ifnar1* ^*-/-*^) and on day 9 after mock (n = 5 WT, n = 4 *Ifnar1* ^*-/-*^) and PR8 infection (n = 5 WT, n = 4 *Ifnar1* ^*-/-*^). Copy numbers of *Enterobacteriaceae* per μl of fecal microbial DNA is shown. Each dot represents one mouse, the geometric mean is indicated. P values were calculated by One-Way ANOVA (Bonferroni multiple comparison test). ***p < 0.001; ns, not significant. One representative experiment is shown. Abbreviations are as follows: Uncl., unclassified; uninf, uninfected.

Mice were monitored daily for 17 days after infection. We assessed the microbiota composition in the fecal content of WT and *Ifnar1* ^*-/-*^ mice before PR8 or mock infection and at 9 day post infection (dpi) (Fig 1A) since the peak weight loss was observed at 9 dpi in WT and *Ifnar1* ^−/−^ mice. PR8 viral load was quantified after non-surgical intratracheal instillation at 1 dpi, and we detected live virus only in the lungs, neither in the colon content nor in the cecum tissue (S1A and S1B Fig). MiSeq Illumina analysis of microbial DNA extracted from fecal samples confirmed observations reported by others [18] that the mouse intestinal microbiota, independent of the genotype, consists of two major bacterial phyla, the Bacteroidetes and the Firmicutes (Fig 1B), with the most relevant classes being Bacteroidia and Clostridia (Fig 1C). No statistical differences were found in the fecal microbiota composition between WT and *Ifnar1* ^*-/-*^ mice, either before infection at day 0 or after mock infection at day 9. Moreover, we observed low abundance of Proteobacteria in the intestinal microbiota of the uninfected and mock-infected mice, previously reported by others [19], independent of the mouse genotype (Fig 1B). Furthermore, at day 9 post PR8 infection, Bacteroidetes and Firmicutes were still the most dominant colonizers in both mouse genotypes (Fig 1B). Our findings, however, uncovered a significant blooming of Proteobacteria at day 9 after PR8 infection only in the WT mice, whereas no significant increase was noted in the *Ifnar1* ^*-/-*^ mice, irrespective of the infection (Fig 1B). Indeed, while Proteobacteria represented 1% on average in uninfected and mock-infected mice, regardless of the genotype, they comprised approximately 15% of the total fecal microbiota in the PR8-infected WT mice (p = 0.0340 One-Way ANOVA after Bonferroni correction) (Fig 1B). The most striking change in the fecal microbial community of WT mice after PR8 infection was the increased abundance of the genus *Escherichia*, being however mostly undetectable in uninfected and mock-infected mice of both genotypes (p = 0.0011 One-Way ANOVA after Bonferroni correction) (Fig 1C).

Overall, greater Proteobacteria colonization levels after influenza infection in WT mice were not caused by differences in Proteobacteria abundance between WT and *Ifnar1* ^*-/-*^ mice prior to PR8 infection. Moreover, the thriving of Proteobacteria after PR8 infection in the WT but not *Ifnar1* ^*-/-*^ mice supports our hypothesis that influenza virus is able to alter the intestinal microbiota, and that this action is dependent on IFN-Is. In addition, using 16S quantitative PCR (qPCR) analysis we confirmed a significant increase in *Enterobacteriaceae* in the stool samples of the PR8-infected WT mice, but not in the PR8-infected *Ifnar1* ^*-/-*^ mice (Fig 1D), however no significant difference was found between WT and *Ifnar1* ^*-/-*^ mice at day 0 prior to infection (Fig 1D). Furthermore, we detected a significant lower level of *Segmented Filamentous Bacteria* (*SFB*) in the stool samples of the PR8-infected WT mice compared to the uninfected WT mice (S1C Fig). *SFB* are Clostridia-correlated bacteria closely attached to the intestinal epithelium, which are able to activate a range of host defenses, including the production of antimicrobials, development of Th17 cells and increased colonization resistance to the intestinal pathogen *Citrobacter rodentium* [9]. However, uninfected WT and *Ifnar1* ^*-/-*^ mice were found similarly colonized with *SFB*; furthermore, the *SFB* abundance did not significantly change in the *Ifnar1* ^*-/-*^ mice, despite PR8 infection (S1C Fig).

In summary, our findings indicate that differences in the fecal microbiota between WT and *Ifnar1* ^*-/-*^ mice prior to influenza infection are insufficient to explain the PR8-mediated changes in specific endogenous bacterial population in WT mice.

Similar results, as observed with influenza, were obtained when synthetic stimulators of IFN-Is such as poly I:C (pIC) [20, 21] were administered to WT and *Ifnar1* ^*-/-*^ mice by non-surgical intratracheal instillation at day 0 and at day 2 (S1D Fig). Using 16S qPCR analysis we found a significant increase in *Enterobacteriaceae* at day 4 and day 5 in the fecal samples of the pIC-treated WT mice, but not in the pIC-treated *Ifnar1* ^*-/-*^ mice (S1E Fig). However, lower level of *SFB* was found at day 4 only in pIC-treated WT mice, but not in the pIC-treated *Ifnar1* ^*-/-*^ mice (S1F Fig).

Collectively, our findings highlight a critical role of type I IFN-mediated signaling induced in the lungs during pulmonary influenza infection in predisposing the host to dysbiosis. Our analysis specifically demonstrates a flourishing of resident bacteria belonging to Proteobacteria pathobionts, and a depletion of a subset of indigenous *SFB*.

### Influenza-induced IFN-Is impair control of *S*. Typhimurium during acute colitis

**Fig 2:**
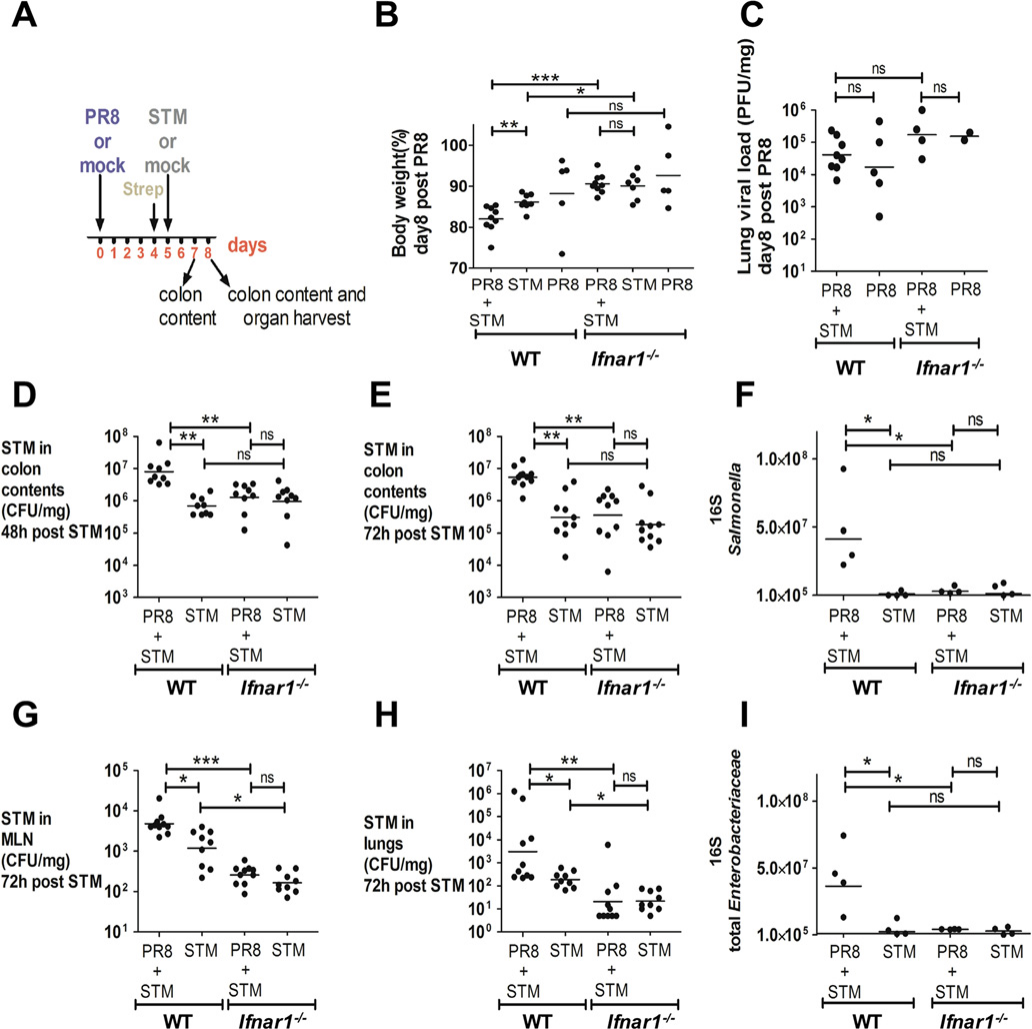
PR8-induced IFN-Is sensitize the host to *S*. Typhimurium infection. A) Schematic representation of the PR8-secondary *S*. Typhimurium infection model. B) Body weight loss at 8 dpi of WT and *Ifnar1* ^*-/-*^ mice in secondarily infected, *S*. Typhimurium-only and PR8-only infected mice. C) Lung viral load was measured at 8 dpi by plaque assay in secondarily infected and PR8-only infected WT and *Ifnar1* ^*-/-*^ mice. D, E) *S*. Typhimurium load in the colon content at 48 h (7 dpi) and 72 h (8 dpi) after bacterial infection. F, I) 16S copy numbers of *Salmonella* (F) and total *Enterobacteriaceae* (I) per μl of microbial DNA from colon content determined at 8 dpi. G, H) *S*. Typhimurium load in MLN and lungs at 72 h after bacterial infection. Each dot represents one mouse, the geometric mean is indicated. *P* values were calculated by two-tailed Mann-Whitney test in (B, D, E, F, G, H, I). Non-parametric Kruskal-Wallis test was used in (C). *p < 0.05, **p < 0.01, ***p < 0.001; ns, not significant. Two independent experiments are shown in (B, D, E, G, H). A representative experiment is shown in (C). A representative experiment is shown in (F, I). N of mice used in each group in (B): PR8 = 5 WT and 5 *Ifnar1* ^*-/-*^, PR8+STM = 9 WT and 9 *Ifnar1* ^*-/-*^, STM = 8 WT and 7 *Ifnar1* ^*-/-*^. N of mice used in each group in (C): PR8 = 5 WT and 2 *Ifnar1* ^*-/-*^, PR8+STM = 8 WT and 4 *Ifnar1* ^*-/-*^. N of mice used in each group in (D, E, G, H): PR8+STM = 9–10 WT and 9–10 *Ifnar1* ^*-/-*^, STM = 9–10 WT and 9–10 *Ifnar1* ^*-/-*^. N of mice used in each group in (F, I): PR8+STM = 4 WT and 4 *Ifnar1* ^*-/-*^, STM = 4 WT and 4 *Ifnar1* ^*-/-*^. Abbreviations are as follows: STM, *S*. Typhimurium.

Since we demonstrated that IFNAR1-mediated signaling increased the abundance of endogenous *Enterobacteriaceae* during influenza infection, we aimed to test whether they could similarly affect the growth of *Salmonella* Typhimurium (*S*. Typhimurium), a leading cause of acute gastroenteritis and inflammatory diarrhea, using a mouse model of acute colitis. One of the hallmarks of *S*. Typhimurium virulence in mice is its systemic manifestations resembling typhoid fever; in the typhoid model no intestinal inflammation is observed, and subsequently *Salmonella* numbers in the colon content are low and extremely variable [22, 23]. To achieve colitis, *S*. Typhimurium must be administered to mice pretreated with the antibiotic streptomycin, this results in its effective colonization of the intestinal lumen, followed by high density growth and mucosal inflammation [22]. In our colitis model, WT and *Ifnar1* ^*-/-*^ mice were treated with streptomycin 1 day prior to *S*. Typhimurium infection in order to achieve acute inflammation of the cecal mucosa. On day 0, mice were first infected with a sublethal dose of PR8 virus or PBS by non-surgical intratracheal instillation, then secondarily infected by oral gavage with 10^7^ CFU of *S*. Typhimurium or given LB medium alone at 5 dpi (Fig 2A). Mice were monitored daily until euthanasia at 8 dpi. At that time point, WT mice infected with PR8, followed by *S*. Typhimurium, referred to as “secondarily infected”, were noted to have significantly more weight loss than those infected only with *S*. Typhimurium (Fig 2B), and no difference in weight loss between the two groups of *Ifnar1* ^*-/-*^ mice was detected at 8 dpi. The extent of the weight loss seen in secondarily infected WT mice at 8 dpi was also greater than in secondarily infected *Ifnar1* ^*-/-*^ group (Fig 2B). Lung viral load measured by plaque assay (Fig 2C) and qPCR (S2A Fig) revealed no difference between the WT and *Ifnar1* ^*-/-*^ groups, implying that the influenza infection was not the direct cause of the weight loss. However, at 7 and 8 dpi, corresponding to 48 and 72 hours (h) post *S*. Typhimurium infection, respectively, we found a significant increase (12-fold and 18-fold, respectively) in the *S*. Typhimurium burden in the colons of the WT mice previously infected with PR8 (Fig 2D and 2E). In contrast, infection with PR8 did not enhance *S*. Typhimurium gut colonization in the *Ifnar1* ^*-/-*^ mice (Fig 2D and 2E). Using 16S qPCR analysis, we also detected a significant increase in the *Salmonella* copy number in the colon content of secondarily infected WT mice at 8 dpi compared to the *S*. Typhimurium-only infected WT mice. No difference was detected in the *Salmonella* gene copies between secondarily infected *Ifnar1* ^*-/-*^ and *S*. Typhimurium-only infected *Ifnar1* ^*-/-*^ mice at 8 dpi (Fig 2F). These results were confirmed by 16S-Denaturing Gradient Gel Electrophoresis (DGGE) analysis performed from the microbial DNA extracted from the colon content 8 dpi (S2B Fig). Likewise, at 8 dpi, a significant increase was noted in the total *Enterobacteriaceae* copy number only in the colon content of secondarily infected WT mice, compared to the *S*. Typhimurium-only infected WT mice (Fig 2I). We interpret this rise in total *Enterobacteriaceae* gene copies to represent an increase in the population of *Salmonella*, since a rise in other *Enterobacteriaceae* was not detected. This is in accordance with previous observations [24] showing no overgrowth of commensal *Enterobacteriaceae*, despite high levels of inflammation, in mice infected with *S*. Typhimurium.

Finally, we enumerated *S*. Typhimurium in the mesenteric lymph nodes (MLN) and lungs of WT and *Ifnar1* ^*-/-*^ mice at 8 dpi. Similar to our findings regarding the colonic burden, prior infection with PR8 enhanced the ability of *S*. Typhimurium to disseminate to the MLN and the lungs in WT but not *Ifnar1* ^*-/-*^ mice (Fig 2G and 2H). Indeed there was significantly less dissemination of *S*. Typhimurium overall in the *Ifnar1* ^*-/-*^ mice compared to WT mice (Fig 2G and 2H).

Similar results in *S*. Typhimurium intestinal colonization were obtained when mice were infected at day 5 with 10^3^ CFU of *S*. Typhimurium, without streptomycin pretreatment (typhoid model) (S2C and S2D Fig). Indeed, as expected, *S*. Typhimurium numbers in the colon content were low and highly variable in *S*. Typhimurium-only infected WT and *Ifnar1* ^*-/-*^ mice, whereas prior infection with PR8 was still able to increase *S*. Typhimurium gut colonization in WT, but not in *Ifnar1* ^*-/-*^ mice (S2D Fig).

Overall, these results further support our hypothesis that PR8 infection predisposes mice to secondary *Salmonella* infection in a IFN-I-dependent manner.

We further tested whether pIC could elicit similar effects in our acute colitis mouse model as shown with influenza. WT and *Ifnar1* ^*-/-*^ mice were intraperitoneally (i.p.) injected with pIC or saline 1 day prior and 2 days after the oral gavage administration of 10^7^ CFU of *S*. Typhimurium or LB alone (S3A Fig). Alternatively, WT and *Ifnar1* ^*-/-*^ mice were treated with pIC through non-surgical intratracheal instillation 1 day prior and 1 day after the oral gavage administration of 10^7^ CFU of *S*. Typhimurium (S4A Fig). *S*. Typhimurium CFUs were measured in the colon content, MLN, lungs and spleen at 72 h post bacterial infection. We found that pIC treatment significantly increased the *S*. Typhimurium burden in the luminal colon of the WT mice, but not *Ifnar1* ^*-/-*^ mice (S3B Fig and S4B Fig). Moreover, we found that pIC enhanced *S*. Typhimurium dissemination in the WT but not in the *Ifnar1* ^*-/-*^ group (S3C–S3E Fig and S4C–S4E Fig). These findings imply that the pro-bacterial effects induced by pIC during *S*. Typhimurium infection are largely mediated by IFN-Is, and that these can potently enhance *S*. Typhimurium pathogenicity.

In summary, our studies have consistently shown that IFN-Is confer a fitness advantage to *Salmonella* in colonizing the intestine and disseminating to systemic sites.

### Influenza-induced IFN-Is inhibit the antimicrobial response in *S*. Typhimurium-induced colitis

We next investigated whether IFN-Is might augment *Salmonella* intestinal colonization and dissemination through the suppression of specific well-characterized antimicrobial genes. To this end, we analyzed the expression of the following: *Ifnγ*, the gene that is of pivotal importance in host defense against intramacrophage pathogens [25], in *Salmonella-*induced colitis [26–28] and in the systemic control of *Salmonella* infections [29, 30]; *S100A9*, the gene that encodes one of the two subunits of calprotectin, an antimicrobial heterodimer that acts as a metal-sequestering protein, which can starve *Salmonella* and many other microorganisms of critical nutrients, such as zinc and manganese [24, 31]; *Lcn2*, the gene that encodes the antimicrobial peptide lipocalin-2, which sequesters iron-laden siderophores to inhibit *Enterobacteriaceae* growth [32]. We and others had already noted that transcript levels of *Ifnγ*, *S100A9*, *and Lcn2* were increased in the ceca of WT streptomycin-pretreated mice during *S*. Typhimurium infection [24, 33].

**Fig 3:**
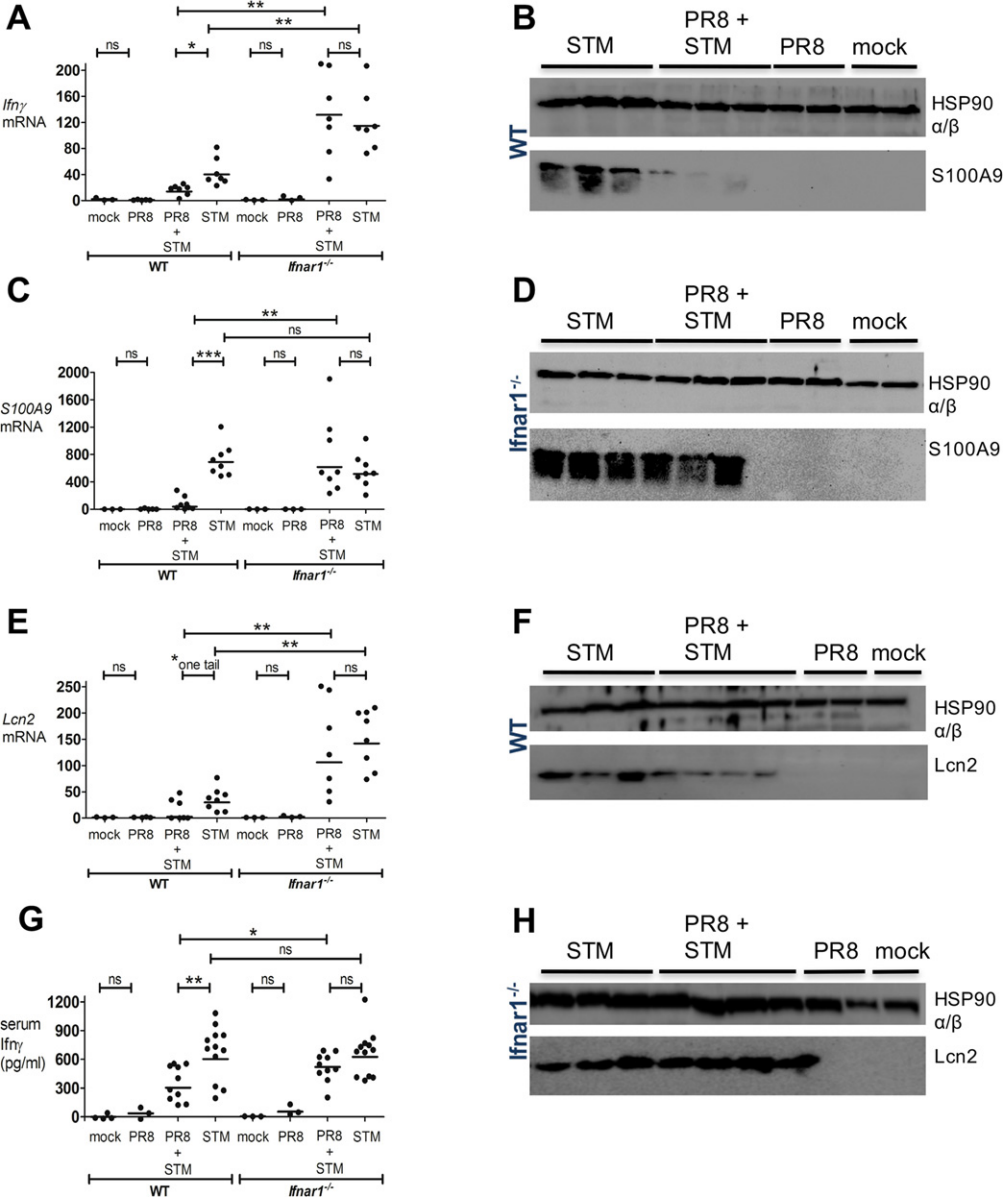
PR8-induced IFN-Is inhibit antimicrobial activity during *S*. Typhimurium infection. WT and *Ifnar1* ^*-/-*^ mice were infected with PR8 or PBS on day 0, followed by intragastric (i.g.) infection with *S*. Typhimurium or LB at 5 dpi. A, C, E) *Ifnγ*, *S100A9* and *Lcn2* transcript levels were detected by qPCR in the mouse cecum of WT and *Ifnar1* ^*-/-*^ at 8 dpi. G) Ifnγ level was detected by ELISA in the serum of WT and *Ifnar1* ^*–/–*^at 8 dpi. B, D) S100A9 and F, H) Lcn2 were detected by immunoblot in the mouse cecum of WT (B, F) and *Ifnar1* ^*-/-*^ (D, H) at 8 dpi. Each dot represents one mouse, the geometric mean is indicated. *P* values were calculated by two-tailed Mann-Whitney test.*p < 0.05, **p < 0.01, ***p < 0.001; ns, not significant. Two independent experiments are shown in A, C, and E. Three independent experiments are shown in G. One representative experiment is shown in B, D, F and H. N of mice used in each group in (A, C, E, G): mock = 3–4 WT and 3 *Ifnar1* ^*-/-*^, PR8 = 3–5 WT and 3 *Ifnar1* ^*-/-*^, PR8+STM = 7–10 WT and 7–10 *Ifnar1* ^*-/-*^, STM = 7–12 WT and 7–12 *Ifnar1* ^*-/-*^. N of mice used in each group in (B, D): mock = 2 WT and 2 *Ifnar1* ^*-/-*^, PR8 = 2 WT and 2 *Ifnar1* ^*-/-*^, PR8+STM = 3 WT and 3 *Ifnar1* ^*-/-*^, STM = 3 WT and 3 *Ifnar1* ^*-/-*.^. N of mice used in each group in (F, H): mock = 1 WT and 1 *Ifnar1* ^*-/-*^, PR8 = 2 WT and 2 *Ifnar1* ^*-/-*^, PR8+STM = 4 WT and 4 *Ifnar1* ^*-/-*^, STM = 3 WT and 3 *Ifnar1* ^*-/-*.^.

We next chose to compare transcript levels in WT and *Ifnar1* ^*-/-*^ mice, which were both previously PR8- or mock-infected, and secondarily infected with *S*. Typhimurium, following the infection model depicted in Fig 2A. At 8 dpi, cecal tissue was excised from the large intestines of WT and *Ifnar1* ^*-/-*^ mice, and transcription of the candidate antimicrobial genes was measured by qPCR. Although basal transcription of *Ifnγ*, *S100A9* and *Lcn2* was similar between mock-infected WT and mock-infected *Ifnar1* ^*-/-*^ mice (Fig 3A, 3C and 3E), the transcription of *Ifnγ and Lcn2* was significantly higher in the ceca of the *Ifnar1* ^*-/-*^ group, compared to the WT group, after infection with *S*. Typhimurium alone (Fig 3A and 3E). Moreover, PR8 infection alone did not change the induction level of these genes in the ceca of WT and *Ifnar1* ^*-/-*^ mice (Fig 3A, 3C and 3E).

However, WT mice secondarily infected with *S*. Typhimurium had a significant reduction in the transcription levels of all three genes, especially *S100A9*, compared to those only infected with *S*. Typhimurium (Fig 3A, 3C and 3E). By contrast, *Ifnar1* ^-/-^ mice showed no difference in the transcript levels of these genes when comparing secondarily *S*. Typhimurium-infected mice with *S*. Typhimurium-only infected mice (Fig 3A, 3C and 3E). Differences were also confirmed in WT mice at the protein level; Western Blot revealed drastically reduced production of S100A9 and Lcn2 in the ceca of the secondarily *S*. Typhimurium-infected mice compared to the *S*. Typhimurium-only infected mice (Fig 3B and 3F). As expected, *Ifnar1* ^*-/-*^ groups showed no difference in the expression of these antimicrobial peptides when comparing the two infection groups (Fig 3D and 3H). The concentration of Ifnγ in the serum of either WT and *Ifnar1* ^*-/-*^mice infected only with PR8 or PBS did not rise above basal levels (36 pg/ml for WT-PR8 and 66 pg/ml for *Ifnar1* ^*-/-*^-PR8-infected groups; Fig 3G). Yet, the serum level of Ifnγ was found drastically reduced in the secondarily infected WT group, in comparison with the *S*. Typhimurium-only infected WT group; by contrast, no disparity was detected between the same groups in the *Ifnar1* ^*-/-*^ cohort (Fig 3G).

Similar effects were observed when the i.p. pIC model instead of the PR8 infection was employed (S5 Fig). S100A9 and Lcn2 were both strongly inhibited at the transcript (S5A and S5B Fig) and protein level (S5E and S5F Fig) in the ceca of WT but not *Ifnar1* ^*-/-*^ mice following pIC treatment then *S*. Typhimurium infection. Cecal *Ifnγ* transcription levels were also reduced by pIC treatment in the WT mice infected with *S*. Typhimurium, but not in the *S*. Typhimurium-infected *Ifnar1* ^*-/-*^ mice (S5C Fig). Moreover, pIC treatment dramatically lowered the Ifnγ serum level in the *S*. Typhimurium-infected WT mice, but not in the *S*. Typhimurium-infected *Ifnar1* ^*-/-*^ mice (S5D Fig).

All together, our findings demonstrate that IFNAR1-mediated signaling can inhibit the host antimicrobial response to *Salmonella* infection.

### Influenza-induced IFN-Is downregulate the inflammatory response in the intestine of mice infected with *S*. Typhimurium

**Fig 4:**
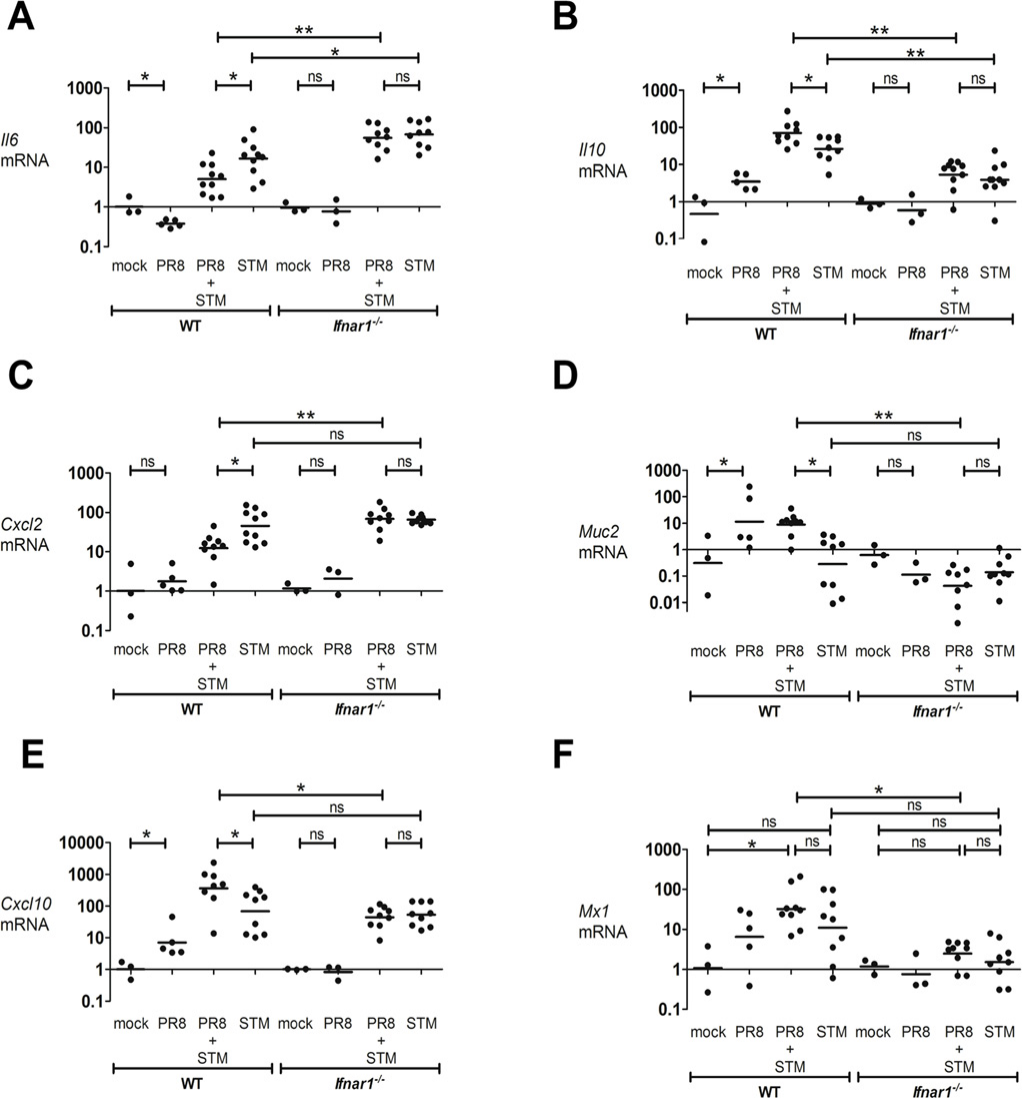
PR8-induced IFN-Is reduce inflammatory response in the gut during *S*. Typhimurium infection. WT and *Ifnar1* ^*-/-*^ mice were infected with PR8 or PBS on day 0, followed by i.g. infection with *S*. Typhimurium or LB at 5 dpi. (A) *Il6*, (B) *Il10*, (C) *Cxcl2*, (D) *Muc2*, (E) *Cxcl10* and (F) *Mx1* transcript levels were detected by qPCR in the cecum of WT and *Ifnar1* ^*-/-*^ mice at 8 dpi. Each dot represents one mouse, the geometric mean is indicated. *P* values were calculated by two-tailed Mann-Whitney test. *p < 0.05, **p < 0.01; ns, not significant. Two independent experiments are shown. N of mice used in each group in (A, B, C, D, E, F): mock = 3 WT and 3 *Ifnar1* ^*-/-*^, PR8 = 5 WT and 3 *Ifnar1* ^*-/-*^, PR8+STM = 8–10 WT and 8–10 *Ifnar1* ^*-/-*^, STM = 8–10 WT and 8–10 *Ifnar1* ^*-/-*^.

To broaden our study of the effects IFN-Is have on the level of cytokine expression in an inflammatory setting such as *Salmonella*-induced colitis, we examined the expression of the pro-inflammatory genes *Il6* and *Cxcl2*, and the anti-inflammatory genes *Il10* and *Muc2*. IL-6 and CXCL2 play important roles in macrophage activation and neutrophil function and recruitment. IL-10 and MUC2 are considered essential immunoregulators in the intestinal tract. IL-10 mainly functions to dampen excessive inflammatory responses that risk damaging the host [34, 35]. MUC2 is critical for colon protection, as *Muc-2* deficient mice spontaneously develop colitis [36, 37]. Their transcript level in the cecum was assessed in both WT and *Ifnar1* ^*-/-*^ mice at 8 dpi after PR8 or mock infection, and following a secondary *S*. Typhimurium infection. After *S*. Typhimurium infection, both pro-inflammatory genes were induced in the cecum of WT and *Ifnar1* ^*-/-*^ mice (Fig 4A and 4C).

Significantly higher transcription of *Il6* was observed in *S*. Typhimurium-only infected *Ifnar1* ^*-/-*^ mice compared to *S*. Typhimurium-only infected WT mice (Fig 4A). Interestingly, in WT but not *Ifnar1* ^*-/-*^ mice, the secondarily infected *S*. Typhimurium group showed significantly lower levels of pro-inflammatory gene transcription than the *S*. Typhimurium-only infected group (Fig 4A and 4C). By contrast, PR8 infection enhanced cecal *Il10* levels in an IFNAR1-dependent manner (Fig 4B). Basal transcript expression of *Il10* was overall similar in mock-infected WT and *Ifnar1* ^*-/-*^ mice. However, we observed a lower level of induction of *Il10* in *S*. Typhimurium-only infected *Ifnar1* ^*-/-*^ mice compared to *S*. Typhimurium-only infected WT mice (Fig 4B). We did not detect upregulation of *Muc2* in *S*. Typhimurium-only infected WT or *Ifnar1* ^*-/-*^ mice, although our samples showed a high level of variability (Fig 4D). However, in WT but not *Ifnar1* ^*-/-*^ mice, PR8 infection enhanced cecal *Muc2* levels. Furthermore, we observed an approximately 10-fold upregulation of *Muc2* in the secondarily infected WT group compared to the *S*. Typhimurium-only infected WT group (Fig 4D).

To further study the contribution of IFN-Is in the modulation of the intestinal host response during *Salmonella* infection, we examined the transcription of *Ifnβ and Ifnα4* in both the lungs and cecum of mice infected with PR8. We found their induction in the lungs but not in the cecum. We also examined the transcription level of *Cxcl10* and *Mx1*, ISGs strongly induced by IFN-Is. Strikingly, we observed approximately 10-fold upregulation of *Cxcl10* in the cecum of PR8-infected WT mice compared to mock-infected WT mice. In contrast, no difference was detected between *Ifnar1* ^*-/-*^ groups (Fig 4E). Moreover, in WT but not *Ifnar1* ^*-/-*^ mice secondarily-infected with *S*. Typhimurium, the level of induction of *Cxcl10* in the cecum was approximately 4-fold higher than *S*. Typhimurium-only infected mice (Fig 4E). Similarly, the cecal expression of *Mx1* was largely upregulated after PR8 infection in the WT mice, but not in the *Ifnar1* ^*-/-*^ mice, and as anticipated the level of induction of *Mx1* tended to be lower overall in the *Ifnar1* ^*-/-*^ mice compared to WT mice (Fig 4F). Remarkably, we observed a 12-fold upregulation of *Mx1* in the secondarily infected WT mice compared to the secondarily infected *Ifnar1* ^*-/-*^ mice (Fig 4F).

In conclusion, the dissimilar regulation of ISGs in the cecum of WT and *Ifnar1* ^*-/-*^ mice after PR8 infection suggest that type I IFN-mediated signaling significantly contributes to the host response against *S*. Typhimurium infection.

**Fig 5:**
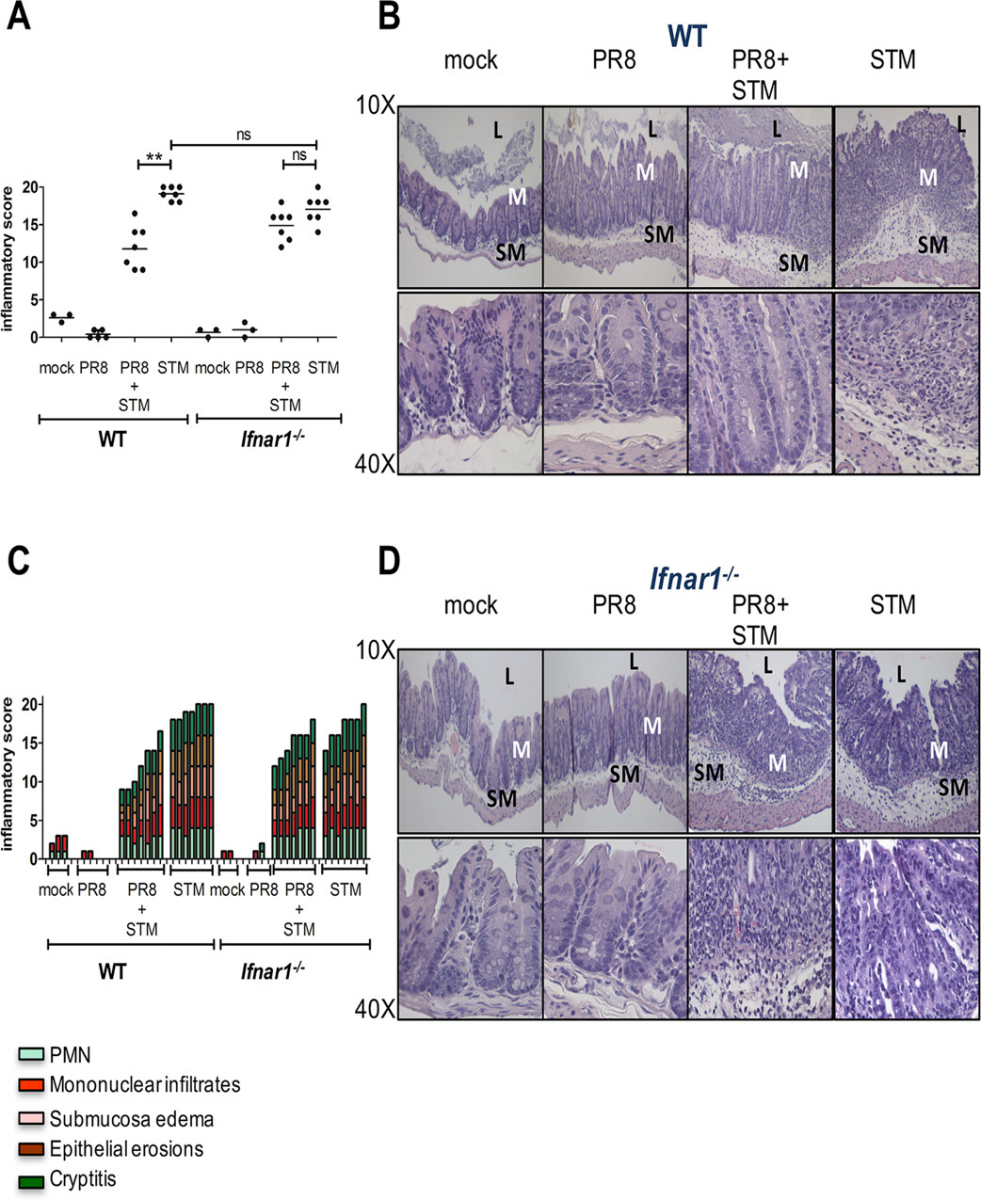
PR8-induced IFN-Is reduce intestinal pathology during *S*. Typhimurium infection. Cecal histopathology of WT (A, B and C) and *Ifnar1* ^*-/-*^ (A, C and, D) mice. Blinded histopathology scores of cecal samples collected at 8 dpi from PR8- and mock-infected WT and *Ifnar1* ^*-/-*^ mice, administered or not with *S*. Typhimurium at 5 dpi. The score of individual mice (circles) and the geometric mean for each group (bars) are indicated in (A). *P* values were calculated by two-tailed Mann-Whitney test. **p < 0.01; ns, not significant. Two independent experiments are shown. N of mice used in each group in (A, C): mock = 3 WT and 3 *Ifnar1* ^*-/-*^, PR8 = 5 WT and 3 *Ifnar1* ^*-/-*^, PR8+STM = 7 WT and 7 *Ifnar1* ^*-/-*^, STM = 7 WT and 7 *Ifnar1* ^*-/-*^. A detailed scoring for the animals shown in (A) is provided; each stacked column represents an individual mouse in (C). B, D) Hematoxylin and eosin (H&E)-stained sections from representative animals for each group in WT (B) and *Ifnar1* ^*-/-*^ (D) mice. An image at lower magnification (10X) and one at higher magnification (40X) from the same section are shown. Abbreviations are as follows: L, lumen; M, mucosa; SM, submucosa.

Histopathology from the cecum extracted at 8 dpi illustrates that WT and *Ifnar1* ^*-/-*^ mice develop severe inflammation 72 h after infection with *S*. Typhimurium, whereas no abnormalities are seen after PR8 or mock infection. However, WT mice infected with PR8 followed by *S*. Typhimurium infection had reduced inflammation compared to WT mice infected with *S*. Typhimurium alone (Fig 5A). Indeed, the mucosal integrity (assessed by cryptitis and epithelial erosions), inflammatory cell infiltration and submucosal edema were exacerbated in the *S*. Typhimurium-only infected WT group compared to secondarily infected WT group (Fig 5B and 5C). However, no difference in the histopathology was noted between *S*. Typhimurium-only infected *Ifnar1* ^*-/-*^ group compared to secondarily infected *Ifnar1* ^*-/-*^group (Fig 5A, 5C and 5D). Similar differences in the cecal inflammatory score detected by histopathology were noted when pretreating WT mice with pIC before *S*. Typhimurium infection (S6A–S6C Fig and S7A–S7C Fig). However, pIC did not reduce the inflammatory score in the cecum of *S*. Typhimurium-infected *Ifnar1* ^*-/-*^ mice (S6A, S6C and S6D Fig; S7A, S7C and S7D Fig).

To summarize, during *S*. Typhimurium-induced colitis, prior infection with influenza alters gut immune response promoting anti-inflammatory cytokines and reducing pro-inflammatory cytokines through an IFNAR1-dependent mechanism. The end result is a decrease, mediated by IFN-Is, of the intestinal tissue damage in mice that were first infected with influenza prior to *S*. Typhimurium infection.

## Discussion

IFN-Is are primarily considered to be antiviral and immunomodulatory cytokines [38]; their effects during bacterial infection are still controversial. Although IFN-Is have been shown to protect against and limit infection with certain bacterial pathogens [39, 40], they can also impair the clearance of others [41, 42]. This suggests a complex and bacterium-specific mechanism of action.

Influenza virus is a major cause of respiratory illness in humans, with the potential to cause lung damage and sensitize the host to secondary pulmonary infections [1]. Although gastroenteritis symptoms have been reported during infection, the mechanism through which influenza virus would affect the gut is not completely clear. Our studies have shown that influenza pulmonary infection has an effect on the mouse fecal microbiota and promotes secondary infection with the intestinal pathogen, *S*. Typhimurium. Importantly, we have shown that these effects are dependent on IFN-Is, which are induced in the lungs during influenza pulmonary infection. These in turn can alter the gut microbial composition and suppress host immunity to a secondary *Salmonella* intestinal infection.

In line with a previous study [6], we found that PR8 infection and poly I:C treatment increased the relative abundance of *Enterobacteriaceae* and decreased the number of *SFB* in the fecal content of WT mice. However, we found that the relative abundance of these bacterial groups remained unchanged after PR8 infection or poly I:C treatment in mice deficient in IFN-I signaling, demonstrating that IFN-Is play an essential role in regulating their numbers. Based on these results and consistent with previous observations [43], we speculate that IFN-Is might regulate the populations of the major bacterial phyla within the intestinal tract. Moreover, our analysis showed that only particular members of the fecal microbiota were affected by PR8-induced IFN-Is. While anaerobes, such as Bacteroidetes and Firmicutes, make up the majority of the healthy microbiota, IFN-Is released during influenza infection promote the blooming of indigenous Proteobacteria pathobionts to the detriment of restricted anaerobic commensals, leading to significant intestinal disbyosis. This is in accordance with previous findings that show that bacterial imbalance characterized by an enrichment of Proteobacteria is observed during intestinal inflammatory disorders in humans, including Crohn's disease [44] and enteropathy in human immunodeficiency virus (HIV)-infected subjects [45].

In addition, we show that influenza- and poly I:C-induced IFN-Is promote the intestinal overgrowth of the bacterial pathogen *S*. Typhimurium, which is an important component of *Enterobacteriaceae* and clinically associated with severe gastroenteritis and inflammatory diarrhea in humans [46].

Furthermore, we showed that *Ifnar1* ^*-/-*^ mice were more resistant to weight loss during *S*. Typhimurium secondary infection than WT mice, and allowed less translocation of *S*. Typhimurium across the intestinal wall. Not surprisingly, we also reported an increased resistance in *Ifnar1* ^*-/-*^ mice against single *S*. Typhimurium infection, as indicated by reduced weight loss and bacterial dissemination, as well as higher induction of specific antibacterial genes, compared to *S*. Typhimurium-only infected WT mice. This is in agreement with previous reports where *S*. Typhimurium has been shown to induce the expression of IFN-Is by macrophages [47], along with improved host survival and enhanced control of *S*. Typhimurium in *Ifnar1* ^*-/-*^ mice in a necroptosis-dependent mechanism [48]. While there were differences between *S*. Typhimurium-only infected WT and *Ifnar1* ^*-/-*^ mice, these differences were strongly exacerbated when mice were previously infected with PR8, indicating the contribution of IFN-Is produced during influenza infection to secondary *S*. Typhimurium infection.

Considering that the number of *S*. Typhimurium that disseminated systemically was greatly increased following influenza infection in WT, but not in *Ifnar1* ^*-/-*^ mice, this suggests that IFN-Is may potentially relax the intestinal barrier to allow for *Salmonella* systemic dissemination. In support of this, we found that the host inflammatory and antimicrobial responses against *S*. Typhimurium were reduced in the intestine after influenza infection in a mechanism dependent on IFNAR. Host inflammatory responses are essential to keep *Salmonella* localized to the gut and to limit the systemic spread of the pathogen. The immunosuppression mediated by IFN-Is would diminish local host surveillance, resulting in poor control of *Salmonella* in the gut and enhanced bacterial dissemination. We demonstrated that the induction of IFN-γ was decreased at the transcriptional level in the inflamed cecum and at the protein level systemically in an IFN-I-dependent manner. This is important given the key role IFN-γ plays in intestinal antibacterial immunity, host survival and resolution of *Salmonella* infection [49].

The host inflammatory response against *Salmonella* in the gut [50] includes the synthesis of antimicrobial peptides, some of which may possess a secondary function as regulatory molecules [51]. Two critical components of the mammalian nutritional immune response against *S*. Typhimurium are lipocalin-2 and calprotectin, which are both highly induced by this pathogen in the inflamed gut. Lipocalin-2 and calprotectin sequester essential nutrients from microorganisms and exert an antimicrobial effect against several bacteria, including intestinal commensals. Both antimicrobials were dramatically suppressed in the inflamed gut after PR8 infection in a mechanism that depended on IFNAR signaling. Paradoxically, we know that the metal deficiency induced by high levels of both antimicrobials can be evaded by *Salmonella* through expression of high-affinity metal transporters [24, 52]. However, metal starvation may still have a critical defensive role against *Salmonella* by forcing infected host cells in the local microenvironment to undergo apoptosis [53, 54], thereby destroying the internalized *Salmonella*. Furthermore, both antimicrobial peptides act as crucial paracrine chemoattractants to recruit neutrophils [55, 56], which play a major role in preventing systemic dissemination of *S*. Typhimurium, as suggested by clinical and experimental data [57]. Additionally, the induction in the gut of anti-inflammatory *Il10* after influenza infection might, not only inhibit the bactericidal response of macrophages, but also cause infected macrophages to function as hosts for bacterial replication, as previously shown [58]. IL-10 has been shown to be important in the pathogenesis of *Salmonella* infection and regulation of subsequent host immune responses. IL-10 levels are elevated in susceptible strains of mice [59] suggesting that those strains producing IL-10 at high levels cannot adequately control *Salmonella* infection. Moreover, the relevance of the anti-inflammatory role mediated by IFN-Is in the gut during *Salmonella* infection was confirmed by histopathology, which underscores the concept of the double-edged sword IFN-Is represent during secondary intestinal infections. Although IFN-I-mediated effects promote *Salmonella* intestinal colonization and systemic dissemination, they also limit the damage triggered by exacerbated inflammation induced by *Salmonella* infection. Furthermore, several clinical trials could not completely define the therapeutic effects of IFN-Is in patients with ulcerative colitis, an IBD with a complex etiology that includes genetic and environmental factors leading to chronic inflammatory responses against the gut microbiota [60, 61]. This raises important questions about the potential mechanisms of action of IFN-Is in IBDs such as ulcerative colitis. Notably, IFNβ therapy markedly attenuates the course and severity of disorders such as Multiple Sclerosis (MS). Indeed, *in vitro* studies previously showed that IFNβ induced the release of the anti-inflammatory cytokine IL-10 from lymphocytes acquired from patients with MS [62, 63], which might indicate that IFNβ could eventually induce an anti-inflammatory response in the colonic mucosa as well.

Many investigators have reported a variety of both beneficial and detrimental immune functions for IFN-Is during bacterial infections, and this clearly expands the old misguided notion that IFN-Is serve “only” as antiviral cytokines. Specifically, we have examined the effects of IFN-Is induced by pulmonary influenza infection in intestinal bacterial homeostasis and during secondary enteric infection. We propose that during influenza infection, this family of cytokines alters the intestinal microbiota composition, leading to an overgrowth of pathobionts which puts the host at risk to develop intestinal bacterial disorders. This is particularly significant as IFN-Is are currently being used as anti-inflammatory therapies for several immunological disorders such as IBD and MS.

Furthermore, we propose that influenza-induced IFN-Is enhance susceptibility to *Salmonella* intestinal colonization and dissemination during secondary *Salmonella*-induced colitis through suppression of host intestinal immunity.

Our work highlights the critical importance of further studies that clarify the roles and effects IFN-Is play in balancing host susceptibility to bacterial infection and inflammatory control, as well as the potential risk associated with influenza infection in predisponing the host to *Salmonella* infections and intestinal disorders.

## Materials and Methods

### Bacterial strain and culture conditions

IR715 is a fully virulent, nalidixic acid-resistant derivative of *Salmonella enterica* serotype Typhimurium WT isolate ATCC 14028 [64]. The strain was cultured aerobically in Luria Bertani (LB) broth at 37°C. Bacterial strains and plasmids used in this study are listed in S1 Table.

Carbenicillin was added to final 100 mg/L as needed. To render all strains equally resistant to streptomycin, pHP45omega plasmid [65] was introduced by electroporation.

### Mouse experiments

7–9 week old mice with a C57BL/6J genetic background were used in all experiments. *Ifnar1* ^*−/−*^mice were generated as previously reported [42]. The same number of males and females were used in each treatment group. Colonies of *Ifnar1* ^*−/−*^ and WT mice were maintained and housed in the same pathogen-free facilities at UCLA. The mouse studies described in this manuscript were performed under the written approval of the UCLA Animal Research Committee (ARC) in accordance to all federal, state, and local guidelines. All studies were carried out under strict accordance to the guidelines in The Guide for the Care and Use of Laboratory Animals of the National Institutes of Health and the accreditation and guidelines of the Association for the Assessment and Accreditation of Laboratory Animal Care (AALAC) International under UCLA OARC Protocol Number 2009-012-21.

### Viral mouse infection and lung collection

Mice were infected with a sublethal dose (200 PFU) of the mouse-adapted influenza A/Puerto Rico/8/34 (PR8) virus strain by non-surgical intratracheal instillation [66].

Briefly, mice were anesthetized with a mixture of ketamine and xylazine (100 mg/10 mg/kg), suspended by an incisor wire on an angled stand, and then a fixed volume containing 200 PFU of PR8 in pharmaceutical grade PBS was instilled inside the trachea [66]. Following aspiration of the inoculum into the lungs, mice were maintained on warming pads and monitored until completely ambulatory. Mice were monitored daily and weight was recorded. On day 1, 8 or 17 after PR8 (or mock) infection, mice were euthanized by CO_2_ asphyxiation. Lungs were excised, lobes were separated and placed in a 2 mL FastPrep homogenization tube containing lysing matrix D. Sterile DPBS was added to give a final 20% w/v suspension and homogenized using a FastPrep-24 Instrument, 6 m/s, 45 s (MP Biomedicals, Santa Ana, CA). Lung homogenate was diluted 1:10 in TRIzol Reagent (Life Technologies, Grand Island, NY) for analysis of gene expression, or serially diluted with DPBS for quantification of bacterial burden by CFU analysis and viral titer by plaque assay.

### Viral titering

Viral titer was determined by plating a monolayer of MDCK cells in 6-well tissue culture treated plates, then incubating with lung homogenate, serially diluted in virus dilution buffer (PBS with 1% Pencillin/Streptomycin, 0.2% BSA, 0.005% DEAE Dextran, 1X CaCl_2_/MgCl_2_), for 1 h at 37°C. Extracellular virus was removed by gently washing the monolayer, then an overlay containing 2% low melting point agarose in virus growth medium (MEM containing BME vitamins, 10 mM HEPES, 1% Pencillin/Streptomycin, 0.15% NaHCO_3_, 0.2% BSA, 0.0015% DEAE Dextran, 0.7 mg/ml TPCK-treated Trypsin) was applied and plates were incubated for 2 days at 37°C. The overlay was gently aspirated, then the plates were incubated with 0.3% crystal violet in 20% ethanol and plaque forming units (PFU) were enumerated.

### Analysis of the fecal microbiota after influenza infection

Mice were administered 200 PFU of PR8 virus or PBS through non-surgical intratracheal instillation [66].

Mice were monitored daily at the same time until day 17, when all the mice were euthanized.

Fecal samples were collected from WT and *Ifnar1* ^*−/−*^ mice before PR8 or mock infection on day 0 and at 9 dpi, then snap frozen in liquid nitrogen. The fecal DNA was subsequently extracted using the QIAamp DNA stool kit (Qiagen), according to the manufacturer’s instructions. The fecal microbial DNA was used for 16S quantitative real-time PCR (qPCR) analysis and for Illumina MiSeq analysis.

Two μl of extracted fecal bacterial DNA was used as a template for 16S qPCR reaction with the primer pairs previous developed and presented in S2 Table. The 16S gene copy numbers per μl of DNA from each sample (one fecal pellet collected from each mouse) was determined using the plasmids described in S1 Table.

For MiSeq analysis, bacterial DNA was amplified by a two-step PCR enrichment of the 16S rDNA (V4 region) encoding sequences from each sample with primers 515F and 806R modified by addition of barcodes for multiplexing. Libraries were sequenced using an Illumina MiSeq system. Following quality filtering, the sequences were demultiplexed and trimmed before performing sequence alignments, identification of operational taxonomic units (OTU), clustering, and phylogenetic analysis using QIIME open-source software (http://qiime.org).

### Secondary *S*. Typhimurium infection

Mice were infected on day 0 with 200 PFU of PR8 influenza strain or PBS through non-surgical intratracheal instillation [66]. WT and *Ifnar1* ^*-/-*^ mice were orally gavaged with 0.1 ml of a 200 mg/ml streptomycin/sterile water solution on day 4, prior to mock infection in LB or oral infection with 1×10^7^ CFU of *S*. Typhimurium in LB on day 5. Colon content was collected at 48 h postbacterial infection, weighed, homogenized in 1 ml of sterile PBS, serial diluted and plated on LB agar containing appropriate antibiotics. At 72 h post-bacterial infection the cecum was harvested for mRNA, protein, and histopathology. The colon contents, spleen, lungs and mesenteric lymph nodes (MLN) were collected, serially diluted, and plated on appropriate antibiotic LB agar plates to determine bacterial counts. Lungs were harvested for mRNA isolation and plaque assay. Blood was collected by cardiac puncture, allowed to clot at room temperature; serum was isolated by centrifugation, transferred to a sterile tube and stored at -80°C until ELISA cytokine analysis. Groups of 4–6 mice were used for each experiment. Mouse weight was taken daily until euthanasia.

For the typhoid model, WT and *Ifnar1* ^*-/-*^ mice were infected on day 0 with 200 PFU of PR8 influenza strain or PBS through non-surgical intratracheal instillation [66]. Mice were gavaged with 1×10^3^ CFU of *S*. Typhimurium in LB, without streptomycin pre-treatment, on day 5. The colon contents were collected at 72h post bacterial infection, serially diluted, and plated on appropriate antibiotic LB agar plates to determine bacterial counts.

### Analysis of the fecal microbiota after poly I:C treatment

Polyinosinic polycytidylic acid (#tlrl-pic) was purchased from Invivogen (San Diego, CA). Mice were administered 50 μg of pIC or saline through non-surgical intratracheal instillation [66] on day 0 and on day 2. Fecal samples were collected from WT and *Ifnar1* ^*−/−*^ mice on day 0 before treatment and on day 4 and day 5, then snap frozen in liquid nitrogen. The fecal DNA was subsequently extracted using the QIAamp DNA stool kit (Qiagen), according to the manufacturer’s instructions. The fecal microbial DNA was used for 16S qPCR analysis, as described above, and the copy numbers’ fold increase from each mock and pIC-treated sample (one fecal pellet collected from each mouse) on day 4 and day 5 over the baseline before treatment on day 0 were calculated.

### Poly I:C treatment during *S*. Typhimurium infection

In the i.p. pIC model, WT and *Ifnar1* ^*-/-*^ mice were intraperitoneal injected with 150 μg of pIC or saline on day -1, and intragastrically treated with streptomycin (0.1 ml of a 200 mg/ml solution in sterile water) [22] on day -1. Alternatively, in the non-surgical intratracheal instillation pIC model, WT and *Ifnar1* ^*-/-*^ mice were injected with 50 μg of pIC or saline on day -1, and intragastrically treated with streptomycin (0.1 ml of a 200 mg/ml solution in sterile water) [22] on day -1. In both models, mice were then orally gavaged with a dose of 10^7^ CFU of *S*. Typhimurium in 0.1 ml of LB on day 0 or mock-infected. A booster dose of 100 μg or 50 μg of pIC was administered on day 2 or on day 1 in the i.p pIC or in the non-surgical intratracheal instillation pIC models, respectively.

On day 3, corresponding to 72 h post *S*. Typhimurium infection, mice were euthanized; the cecum was collected for mRNA and protein isolation and also for histopathological analysis. Serum was separated from blood and collected for ELISA cytokine detection. CFU was enumerated from homogenates of colon content, MLN, lungs and spleen serially diluted and plated on agar plates containing the appropriate antibiotic selection.

### Quantitative real-time PCR

Total RNA was extracted from mouse cecal tissue with TRIzol Reagent (Life Technologies). Reverse transcription of 1 μg of total RNA was performed with the iScript cDNA Synthesis kit (Bio-Rad). qPCR was performed using iTaq Universal Sybr Green Supermix (Bio-Rad). For analysis, target gene expression of each sample was normalized to the respective level of *L32* mRNA. Fold changes in gene expression values were then calculated using the mean from the control samples as a baseline and determined using the *ΔΔ* Ct method. A list of qPCR primers used in this study is provided in S2 Table. See also S1 References.

### Murine cytokine ELISA

Mouse IFNγ ELISA Ready-SET-Go! was purchased from eBioscience. Cytokine serum levels were measured according to the manufacturer’s instructions.

### Immunoblot

Total protein was extracted from mouse cecum tissue using TRIzol Reagent (Life Technologies, Grand Island, NY). 15 μg of total protein was resolved using 12.5% SDS-PAGE gels and transferred to PVDF membranes. The membranes were blocked with 2% nonfat dried milk and incubated at 4°C with primary antibodies. Detection of mouse HSP90 α/β was performed with primary rabbit polyclonal antibodies (Santa Cruz Biotechnology), while detection of S100A9 was performed with polyclonal goat anti-mouse S100A9 (R&D Systems). Lcn-2 was detected by polyclonal goat anti-mouse Lcn2 (R&D Systems). After overnight incubation at 4°C, the blots were washed and then incubated for 1 h at room temperature with secondary goat anti-rabbit and donkey anti-goat antibodies conjugate to horseradish peroxidase (HRP) (Southern Biotech and Santa Cruz Biotechnology, respectively). After washing, bands were developed using the SuperSignal West Pico Chemiluminescent Sustrate (Thermo Scientific) per manufacturer’s instructions and visualized using Gel Doc (BioRad).

### PCR-DGGE analysis

Total genomic bacterial DNA was isolated using the MasterPure DNA purification kit (Epicentre). DNA quality and quantity were determined with a Spectronic Genesys UV spectrophotometer at 260 nm and 280 nm (Spectronic Instruments, Inc. Rochester, NY). Amplification of bacterial 16S rDNA was carried out by PCR as described previously [67]. Briefly, the universal primer set Bac1 and Bac2 [68] (S2 Table) was used to amplify an approximately 300-base-pair internal fragment of the 16S rDNA. Each 50 μl PCR contained 100 ng purified genomic DNA, 40 pmole each primer, 200 μM of each dNTP, 4.0 mM MgCl_2_, 5 μl 10 X PCR buffer, and 2.5 U Taq DNA polymerase (Invitrogen). Cycling conditions were 94°C for 3 min, followed by 30 cycles of 94°C for 1 min, 56°C for 1 min and 72°C for 30 s, with a final extension period of 5 min at 72°C. The resulting PCR products were evaluated by electrophoresis through 1.0% agarose. DGGE was performed by use of the Bio-Rad DCode System (Hercules, CA, USA). A 40% to 60% linear DNA denaturing gradient (100% denaturant is equivalent to 7 M urea and 40% de-ionized formamide) was formed in 8% (w/v) polyacrylamide gels. Approximately 300 ng PCR product was applied per lane. The gels were submerged in 1 X TAE buffer (40 mM Tris base, 40 mM glacial acetic acid, 1 mM EDTA) and the PCR products were separated by electrophoresis for 17 h at 58°C using a fixed voltage of 60 V. After electrophoresis, the DNA bands were stained with 0.5 μg/ml ethidium bromide and DGGE profile images were digitally recorded using the Molecular Imager Gel Documentation system (Bio-Rad). DIVERSITY DATABASE Software (Bio-Rad) was used to assess the change in the relative intensity of bands corresponding to bacterial species of interest.

### Identification of bacterial species from DGGE gels

The DNA bands of interest were excised from the DGGE gels and transferred to a 1.5-ml microfuge tube containing 20 μl sterile ddH_2_O. Tubes were incubated at 4°C overnight before the recovered DNA samples were re-amplified with the universal primer set Bac1 and Bac2. The PCR products were purified using the QIAquick PCR purification kit (Qiagen) and sequenced at the UCLA Core DNA Sequencing Facility. The sequences obtained were subjected to nucleotide BLAST searches against the NCBI (http://blast.ncbi.nlm.nih.gov/) and Human Oral Microbiome (http://www.homd.org/index.php) databases.

### Statistics

The differences between treatment groups were analyzed by non-parametric 2-tailed Mann-Whitney U test, non-parametric Kruskal-Wallis test (Dunn's Multiple Comparison Test) and One-Way Analysis of variance (ANOVA) with Bonferroni correction, as specified in each Fig legend. Data were expressed as the geometric mean or mean ± SEM, as indicated in each Fig legend, and the results were considered statistically significant when the p value was < 0.05. All calculations were performed using GraphPad Software, unless indicated otherwise.

### Histopathology

Tissue samples were fixed in formalin for 24 h, processed according to standard procedures for paraffin embedding, sectioned at 4 mm, and stained with hematoxylin and eosin. The pathology score of cecal samples was determined by blinded examinations of cecal sections by a board-certified pathologist using previously published methods [22]. Each section was evaluated for the presence of neutrophils, mononuclear infiltrate, submucosal edema, epithelial erosions and cryptitis. Inflammatory changes were scored from 0 to 4 according to the following scale: 0 = none; 1 = low; 2 = moderate; 3 = high; 4 = extreme. The inflammation score was calculated as a sum of each parameter score and interpreted as follows: 0–3 = within normal limits; 4–8 = mild; 9–14 = moderate; 15–20 = severe.

### Ethics statement

The mouse studies described in this manuscript were performed under the written approval of the UCLA Animal Research Committee (ARC) in accordance to all federal, state, and local guidelines. All studies were carried out under strict accordance to the guidelines in The Guide for the Care and Use of Laboratory Animals of the National Institutes of Health and the accreditation and guidelines of the Association for the Assessment and Accreditation of Laboratory Animal Care (AALAC) International under UCLA OARC Protocol Number 2009-012-21. Influenza infections were performed under ketamine/xylazine anesthesia and all efforts were made to minimize animal pain and discomfort.

## Supporting Information

S1 Fig Changes in the fecal microbiota after PR8 infection and poly I:C treatment are mediated by IFN-Is. A and B) WT and *Ifnar1* ^*-/-*^ mice (n = 4 WT, n = 4 *Ifnar1* ^*-/-*^) were infected with PR8 (n = 3 WT, n = 3 *Ifnar1* ^*-/-*^) or PBS (n = 1 WT, n = 1 *Ifnar1* ^*-/-*^) on day 0 through non-surgical intratracheal instillation. Viral titer was determined by plaque assay in lungs and colon content on day 1 after PR8 infection (A). The levels of the influenza virus–derived matrix M protein gene in both lung and cecum tissues were quantified by qPCR on day 1 after infection (B). C) Analysis of the *Segmented Filamentous Bacteria* (*SFB*) using 16S rRNA gene qPCR from fecal samples collected from mice on day 0 before infection (n = 9 WT, n = 8 *Ifnar1* ^*-/-*^), on day 9 after mock (n = 4 WT, n = 4 *Ifnar1* ^*-/-*^) and PR8 infection (n = 5 WT, n = 4 *Ifnar1* ^*-/-*^). Displayed are copy numbers of *SFB* per μl of fecal microbial DNA. Each dot represents one mouse, the geometric mean is indicated. D, E and F) Mice were treated with pIC (n = 4 WT, n = 3 *Ifnar1* ^*-/-*^) or saline (n = 4 WT, n = 3 *Ifnar1* ^*-/-*^) through non-surgical intratracheal instillation on day 0 and on day 2. Mice were euthanized on day 9. Fecal samples were collected from WT and *Ifnar1* ^*−/−*^ mice on day 0 before treatment and on day 4 and day 5 after treatment (D). Analysis of the fecal *Enterobacteriaceae* (E) and *SFB* (F) using 16S qPCR. Data are expressed as copy numbers’ fold increase of mock- and pIC-treated on day 4 and day 5 over the baseline before treatment on day 0. Data are expressed as mean ± SEM. P values were calculated by Kruskal-Wallis (Dunn’s multiple comparison test) in (C) and by two-tailed Mann-Whitney test in (E and F). *p value < 0.05, **p < 0.01; ns, not significant. One representative experiment is shown. Abbreviations are as follows: n.d., not detected.
(TIF)

S2 Fig Influenza-induced IFN-Is enhance host sensitivity to secondary *S*. Typhimurium infection. A) Lung PR8 was measured by qPCR at 8 dpi, and its relative expression to *L32* was calculated in WT and *Ifnar1* ^*-/-*^ mice that were infected with either PR8-only or secondarily infected with *S*. Typhimurium. One representative experiment is shown. N of mice used in each group in (A): PR8 = 5 WT and 2 *Ifnar1* ^*-/-*^, PR8+STM = 10 WT and 4 *Ifnar1* ^*-/-*^. *P* values were calculated in (A) using non-parametric Kruskal-Wallis test (Dunn’s multiple comparison test). B) Relative abundance of *Salmonella* in colon content of WT (left) and *Ifnar1* ^-/-^ (right) mice revealed by PCR-Denaturing Gradient Gel Electrophoresis (DGGE). Red arrows indicate the *Salmonella* band; mock: mock-infected mice on day 0; PR8: mice infected with PR8 virus on day 0; PR8+STM: mice infected with PR8 virus on day 0, followed by *S*. Typhimurium administration on day 5; STM: mice infected with *S*. Typhimurium on day 5. All the mice illustrated were streptomycin-treated by oral gavage on day 4. The luminal content used to isolate the bacterial DNA was extracted at the end of the experiment on day 8. C) Schematic representation of the secondary *S*. Typhimurium infection model in absence of streptomycin pretreatment (typhoid model). WT and *Ifnar1* ^-/-^ mice were previously infected with PR8 or PBS on day 0, then infected with 10^3^ CFU of *S*. Typhimurium or LB alone on day 5. D) *S*. Typhimurium load in the colon content at 72 h (8 dpi) after bacterial infection in the typhoid model. *P* values were calculated by two-tailed Mann-Whitney test.*p < 0.05; ns, not significant. One representative experiment is shown. N of mice used in each group in (D): PR8+STM = 8 WT and 6 *Ifnar1* ^*-/-*^, STM = 8 WT and 6 *Ifnar1* ^*-/-*^.
(TIF)

S3 Fig Poly I:C-induced IFN-Is promote S. Typhimurium intestinal colonization and systemic dissemination (i.p. model). A) Schematic of the i.p. pIC model. B, C, D, E) Colon content, MLN, lungs and spleen were harvested 72 h (day 3) post bacterial infection for enumeration of *S*. Typhimurium. *P* values were calculated by two-tailed Mann-Whitney test. *p value < 0.05, **p value < 0.01; ns, not significant. Data from two indipendent experiments are shown in (B, C, D and E). N of mice used in each group in (B): pIC +STM = 8 WT and 8 *Ifnar1* ^*-/-*^, STM = 8 WT and 8 *Ifnar1* ^*-/-*^. N of mice used in each group in (C, D, E): pIC +STM = 5–6 WT and 5 *Ifnar1* ^*-/-*^, STM = 5–6 WT and 5 *Ifnar1* ^*-/-*^.
(TIF)

S4 Fig Poly I:C-induced IFN-Is promote S. Typhimurium intestinal colonization and systemic dissemination (non-surgical intratracheal instillation model). A) Schematic of the non-surgical intratracheal instillation pIC model. B, C, D, E) Colon content, MLN, lungs and spleen were harvested 72 h (day 3) post bacterial infection for enumeration of *S*. Typhimurium. *P* values were calculated by two-tailed Mann-Whitney test *p value < 0.05, **p value < 0.01; ns, not significant. Data from a representative experiment is shown. N of mice used in each group in (B, C, D, E): pIC +STM = 5 WT and 5 *Ifnar1* ^*-/-*^, STM = 5 WT and 5 *Ifnar1* ^*-/-*^.
(TIF)

S5 Fig Poly I:C-induced IFN-Is inhibit host immunity during S. Typhimurium infection. A, B, C) *S100A9*, *Lcn2* and *Ifnγ* transcript levels were detected by qPCR in the i.p. pIC model from cecum of WT and *Ifnar1* ^*–/–*^mice 72 h post infection. D) Serum Ifnγ protein was assessed by ELISA in the i.p. pIC model from cecum of WT and *Ifnar1* ^*–/–*^mice 72 h post infection. E, F) HSP90α/β, Lcn2 and S100A9 were detected by immunoblot in the i.p. pIC model from cecum of WT (E) and *Ifnar1* ^*–/–*^(F) mice 72 h post infection from a representative experiment. Each dot represents one mouse, the geometric mean is indicated. *P* values were calculated by two-tailed Mann-Whitney test. *p value < 0.05, **p value < 0.01, ***p value < 0.001; ns, not significant. N of mice used in each group in (A, B, C, D): mock = 3 WT and 3 *Ifnar1* ^*–/–*^, pIC = 3–5 WT and 3–5 *Ifnar1* ^*–/–*^, pIC+STM = 7–10 WT and 7–10 *Ifnar1* ^*–/–*^, STM = 7–10 WT and 7–10 *Ifnar1* ^*–/–*^. N of mice used in each group in (E, F): mock = 1 WT and 1 *Ifnar1* ^*–/–*^, pIC = 4WT and 4 *Ifnar1* ^*–/–*^, pIC+STM = 5 WT and 5 *Ifnar1* ^*–/–*^, STM = 3 WT and 3 *Ifnar1* ^*–/–*^. The samples showed were pooled from two independent experiments in (A, B, C and D) or used from one representative experiment in (E, F).
(TIF)

S6 Fig Poly I:C reduces cecal histopathology in WT mice, but not in *Ifnar1* ^*-/-*^ mice during *S*. Typhimurium infection (i.p. model). Blinded histopathology scores of cecal samples from WT (A, B and C) and *Ifnar1* ^*–/-*^ (A, C and, D) mice at 72 h post *S*. Typhimurium or mock infection, i.p. pIC- or mock- treated. The score of individual mice (circles) and the geometric mean for each group (bars) are indicated in (A). *P* values were calculated by two-tailed Mann-Whitney test. **p < 0.01; ns, not significant. One representative experiment is shown. N of mice used in each group in (A, C): mock = 3 WT and 3 *Ifnar1* ^*–/-*^, pIC = 3 WT and 2 *Ifnar1* ^*–/-*^, pIC+STM = 5 WT and 5 *Ifnar1* ^*–/-*^, STM = 5 WT and 5 *Ifnar1* ^*–/-*^. A detailed scoring for the animals shown in (A) is provided; each stacked column represents an individual mouse in (C). B and D) Hematoxylin and eosin (H&E)-stained sections from representative animals for each group in WT (B) and *Ifnar1* ^*-/-*^ (D) mice. Abbreviations are as follows: L, lumen; M, mucosa; SM, submucosa.
(TIF)

S7 Fig Poly I:C reduces cecal histopathology in WT mice, but not in *Ifnar1* ^*-/-*^ mice during *S*. Typhimurium infection (non-surgical intratracheal instillation model). Blinded histopathology scores of cecal samples from WT (A, B and C) and *Ifnar1* ^*–/-*^ (A, C and D) mice at 72 h post *S*. Typhimurium or mock infection, treated or not with pIC through non-surgical intratracheal instillation. The score of individual mice (circles) and the geometric mean for each group (bars) are indicated in (A). *P* values were calculated by two-tailed Mann-Whitney test. *p < 0.05; ns, not significant. One representative experiment is shown. N of mice used in each group in (A, C): mock = 2 WT and 2 *Ifnar1* ^*–/-*^, pIC = 3 WT and 3 *Ifnar1* ^*–/-*^, pIC+STM = 5 WT and 5 *Ifnar1* ^*–/-*^, STM = 5 WT and 5 *Ifnar1* ^*–/-*^. A detailed scoring for the animals shown in (A) is provided; each stacked column represents an individual mouse in (C). B and D) Hematoxylin and eosin (H&E)-stained sections from representative animals for each group in WT (B) and *Ifnar1* ^*-/-*^ (D) mice. Abbreviations are as follows: L, lumen; M, mucosa; SM, submucosa.
(TIF)

S1 Table Strains and plasmids used in this study. (DOCX)

S2 Table Real-time qPCR primers used in this study. (DOCX)

S1 References Supplemental references cited only in the supporting information files. (DOCX)
