# Supplementary figures and images for: Influenza Virus Affects Intestinal Microbiota and Secondary Salmonella Infection in the Gut through Type I Interferons
Source: PLoS Pathog. 2016 May 5;12(5):e1005572. doi: 10.1371/journal.ppat.1005572 (PMC4858270; doi:10.1371/journal.ppat.1005572)

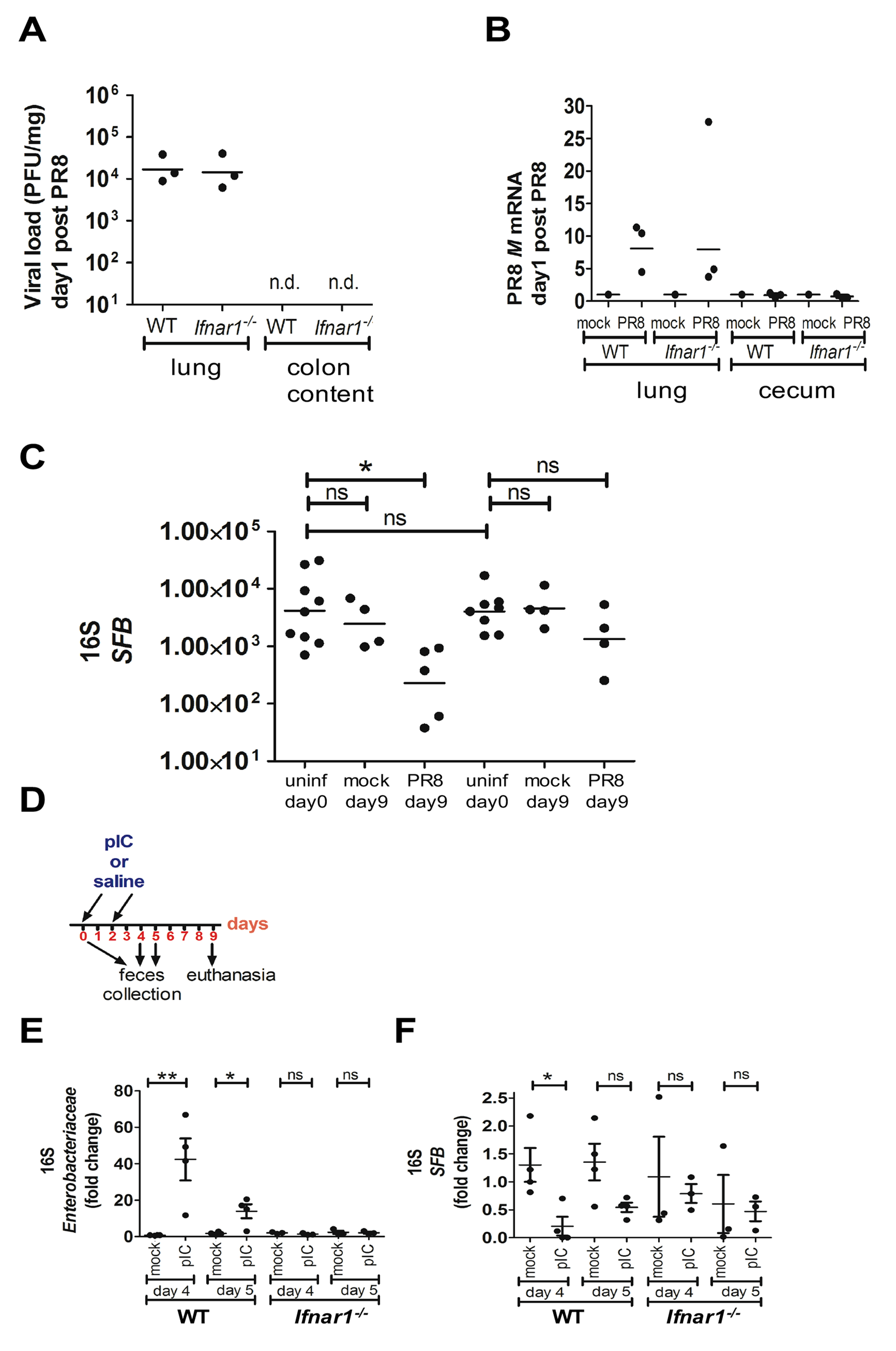

Supplement: S1 Fig — A and B) WT and Ifnar1 -/- mice (n = 4 WT, n = 4 Ifnar1 -/-) were infected with PR8 (n = 3 WT, n = 3 Ifnar1 -/-) or PBS (n = 1 WT, n = 1 Ifnar1 -/-) on day 0 through non-surgical intratracheal instillation. Viral titer was determined by plaque assay in lungs and colon content on day 1 after PR8 infection (A). The levels of the influenza virus–derived matrix M protein gene in both lung and cecum tissues were quantified by qPCR on day 1 after infection (B). C) Analysis of the Segmented Filamentous Bacteria (SFB) using 16S rRNA gene qPCR from fecal samples collected from mice on day 0 before infection (n = 9 WT, n = 8 Ifnar1 -/-), on day 9 after mock (n = 4 WT, n = 4 Ifnar1 -/-) and PR8 infection (n = 5 WT, n = 4 Ifnar1 -/-). Displayed are copy numbers of SFB per μl of fecal microbial DNA. Each dot represents one mouse, the geometric mean is indicated. D, E and F) Mice were treated with pIC (n = 4 WT, n = 3 Ifnar1 -/-) or saline (n = 4 WT, n = 3 Ifnar1 -/-) through non-surgical intratracheal instillation on day 0 and on day 2. Mice were euthanized on day 9. Fecal samples were collected from WT and Ifnar1 −/− mice on day 0 before treatment and on day 4 and day 5 after treatment (D). Analysis of the fecal Enterobacteriaceae (E) and SFB (F) using 16S qPCR. Data are expressed as copy numbers’ fold increase of mock- and pIC-treated on day 4 and day 5 over the baseline before treatment on day 0. Data are expressed as mean ± SEM. P values were calculated by Kruskal-Wallis (Dunn’s multiple comparison test) in (C) and by two-tailed Mann-Whitney test in (E and F). *p value < 0.05, **p < 0.01; ns, not significant. One representative experiment is shown. Abbreviations are as follows: n.d., not detected. (TIF) [file ppat.1005572.s001.tif]

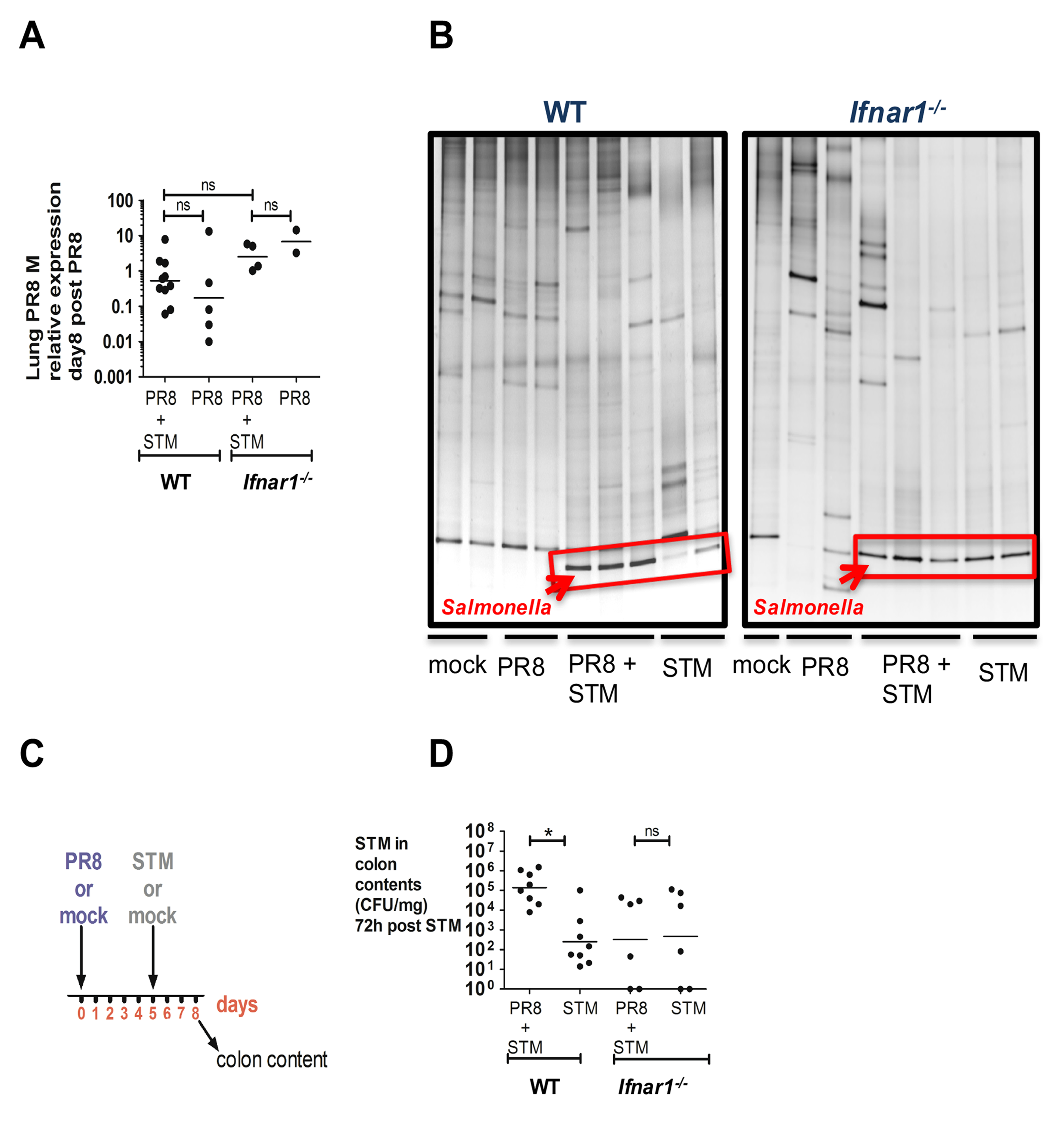

Supplement: S2 Fig — A) Lung PR8 was measured by qPCR at 8 dpi, and its relative expression to L32 was calculated in WT and Ifnar1 -/- mice that were infected with either PR8-only or secondarily infected with S. Typhimurium. One representative experiment is shown. N of mice used in each group in (A): PR8 = 5 WT and 2 Ifnar1 -/-, PR8+STM = 10 WT and 4 Ifnar1 -/-. P values were calculated in (A) using non-parametric Kruskal-Wallis test (Dunn’s multiple comparison test). B) Relative abundance of Salmonella in colon content of WT (left) and Ifnar1 -/- (right) mice revealed by PCR-Denaturing Gradient Gel Electrophoresis (DGGE). Red arrows indicate the Salmonella band; mock: mock-infected mice on day 0; PR8: mice infected with PR8 virus on day 0; PR8+STM: mice infected with PR8 virus on day 0, followed by S. Typhimurium administration on day 5; STM: mice infected with S. Typhimurium on day 5. All the mice illustrated were streptomycin-treated by oral gavage on day 4. The luminal content used to isolate the bacterial DNA was extracted at the end of the experiment on day 8. C) Schematic representation of the secondary S. Typhimurium infection model in absence of streptomycin pretreatment (typhoid model). WT and Ifnar1 -/- mice were previously infected with PR8 or PBS on day 0, then infected with 103 CFU of S. Typhimurium or LB alone on day 5. D) S. Typhimurium load in the colon content at 72 h (8 dpi) after bacterial infection in the typhoid model. P values were calculated by two-tailed Mann-Whitney test.*p < 0.05; ns, not significant. One representative experiment is shown. N of mice used in each group in (D): PR8+STM = 8 WT and 6 Ifnar1 -/-, STM = 8 WT and 6 Ifnar1 -/-. (TIF) [file ppat.1005572.s002.tif]

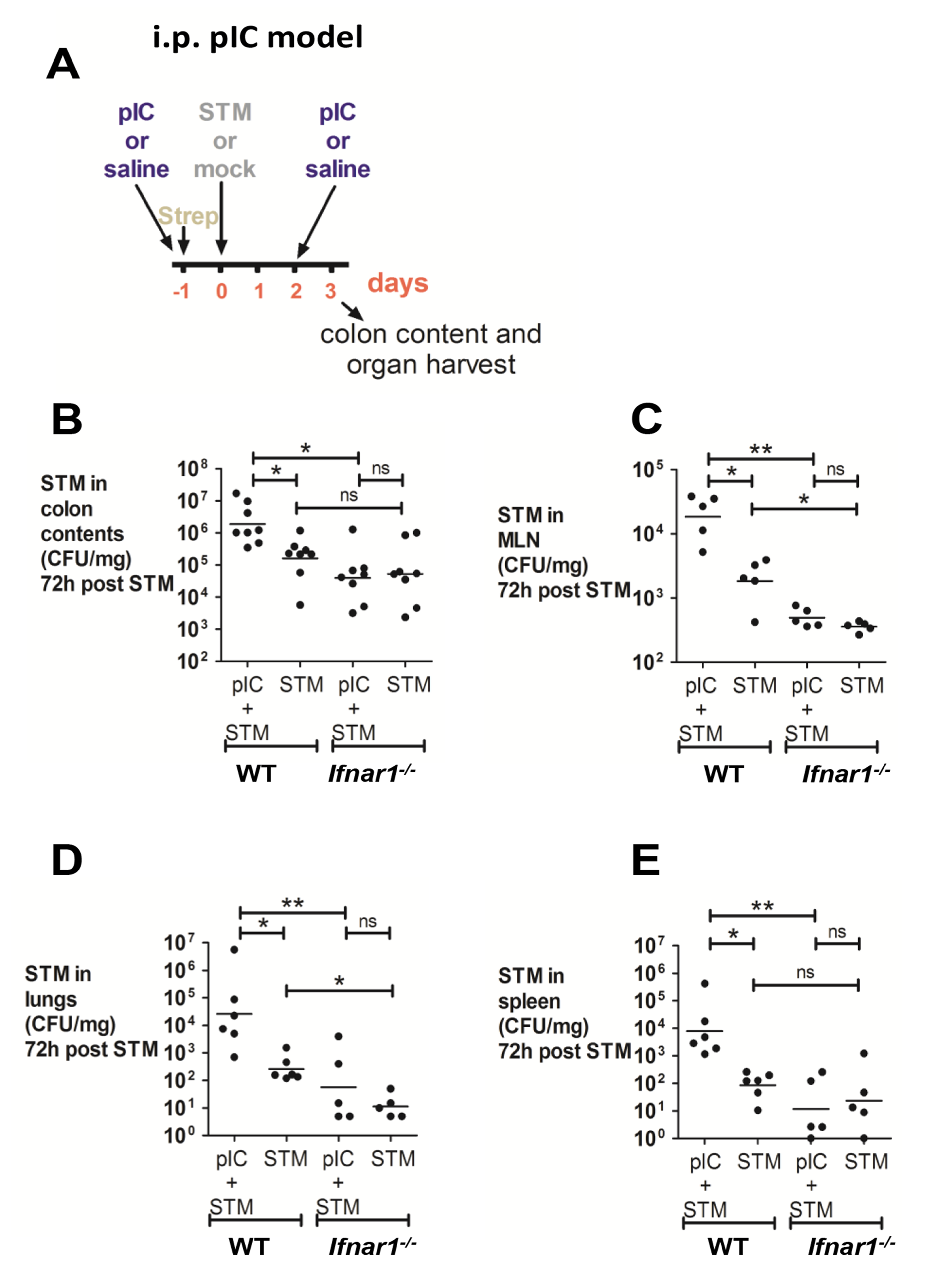

Supplement: S3 Fig — A) Schematic of the i.p. pIC model. B, C, D, E) Colon content, MLN, lungs and spleen were harvested 72 h (day 3) post bacterial infection for enumeration of S. Typhimurium. P values were calculated by two-tailed Mann-Whitney test. *p value < 0.05, **p value < 0.01; ns, not significant. Data from two indipendent experiments are shown in (B, C, D and E). N of mice used in each group in (B): pIC +STM = 8 WT and 8 Ifnar1 -/-, STM = 8 WT and 8 Ifnar1 -/-. N of mice used in each group in (C, D, E): pIC +STM = 5–6 WT and 5 Ifnar1 -/-, STM = 5–6 WT and 5 Ifnar1 -/-. (TIF) [file ppat.1005572.s003.tif]

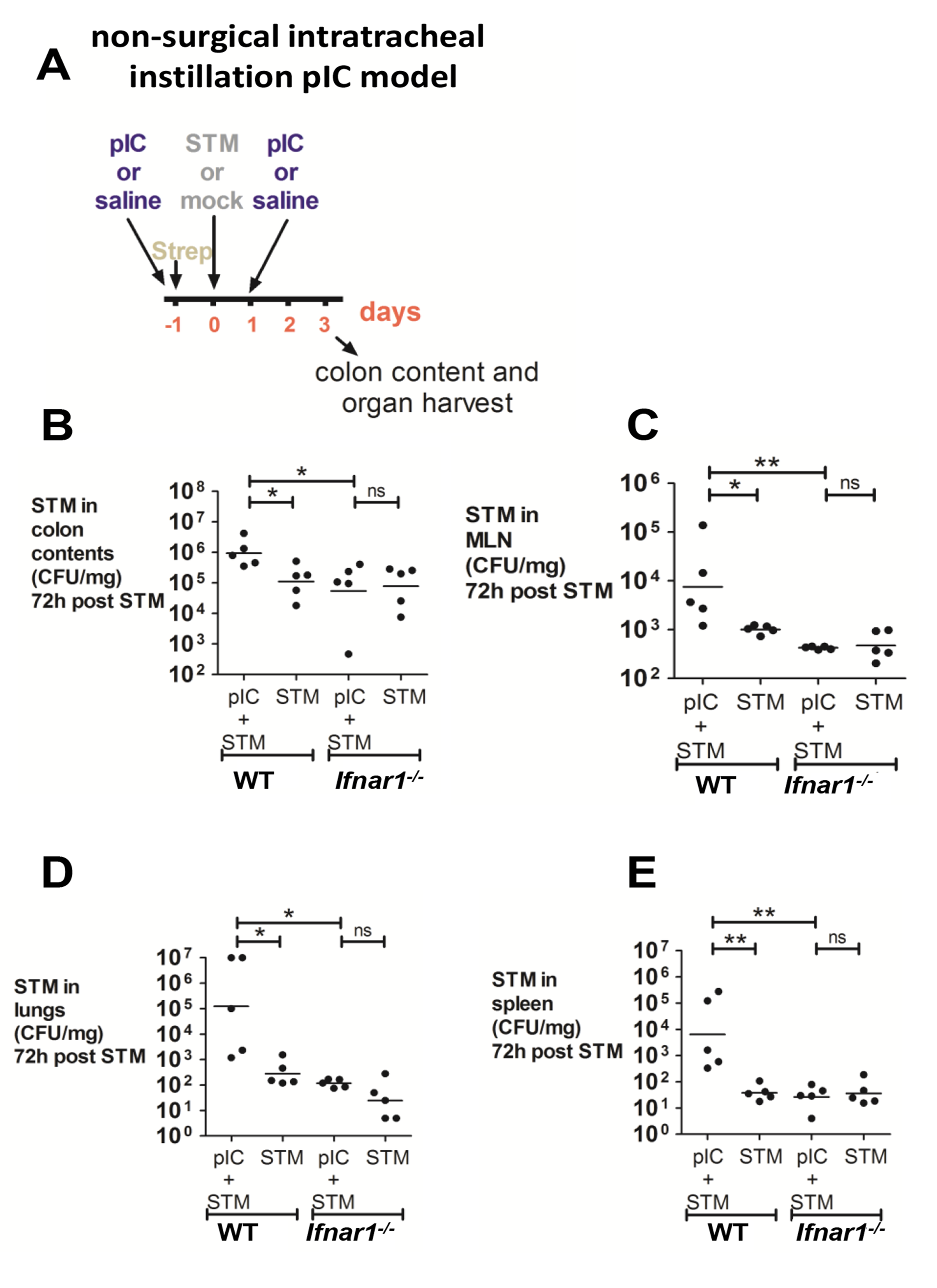

Supplement: S4 Fig — A) Schematic of the non-surgical intratracheal instillation pIC model. B, C, D, E) Colon content, MLN, lungs and spleen were harvested 72 h (day 3) post bacterial infection for enumeration of S. Typhimurium. P values were calculated by two-tailed Mann-Whitney test *p value < 0.05, **p value < 0.01; ns, not significant. Data from a representative experiment is shown. N of mice used in each group in (B, C, D, E): pIC +STM = 5 WT and 5 Ifnar1 -/-, STM = 5 WT and 5 Ifnar1 -/-. (TIF) [file ppat.1005572.s004.tif]

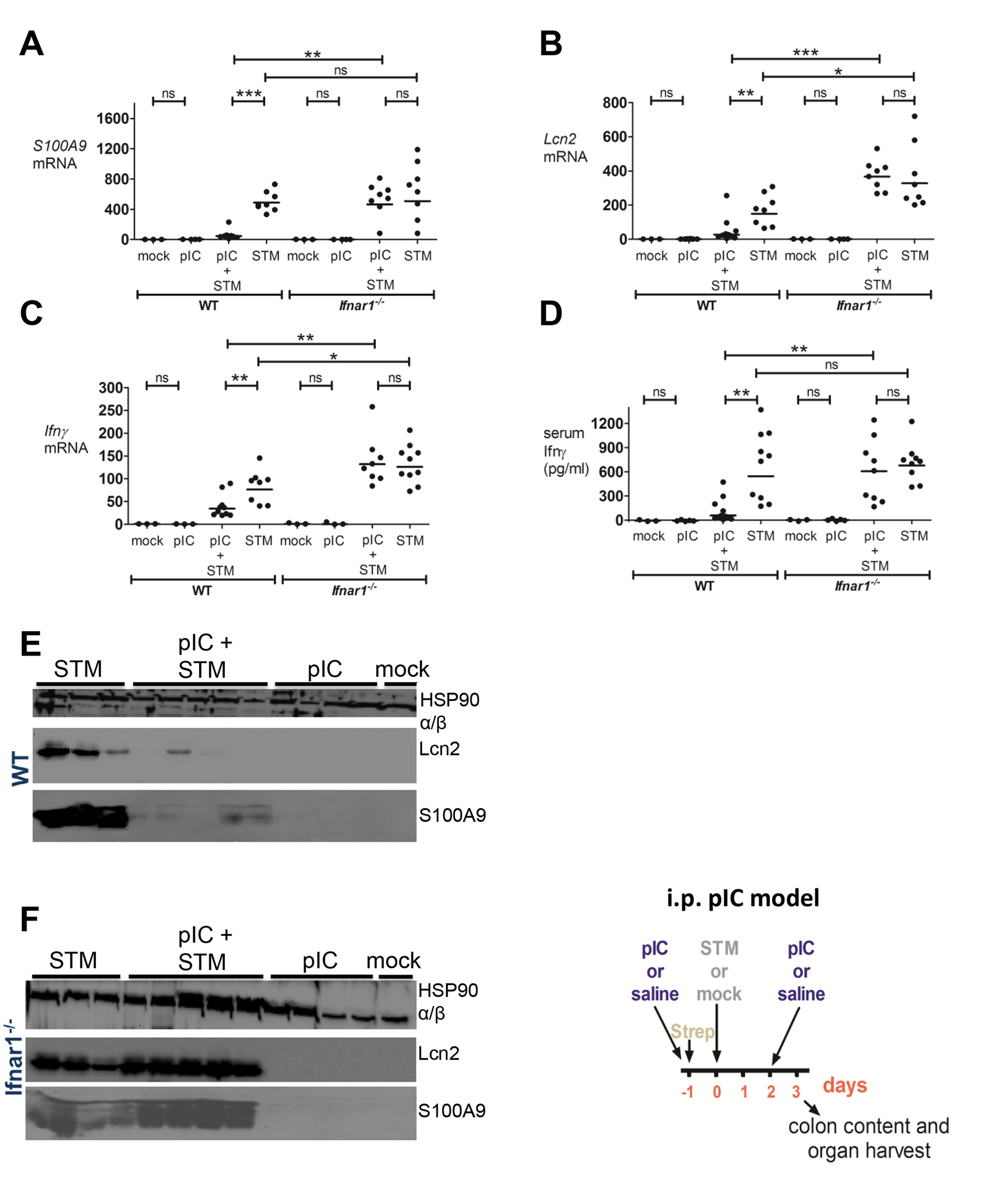

Supplement: S5 Fig — A, B, C) S100A9, Lcn2 and Ifnγ transcript levels were detected by qPCR in the i.p. pIC model from cecum of WT and Ifnar1 –/–mice 72 h post infection. D) Serum Ifnγ protein was assessed by ELISA in the i.p. pIC model from cecum of WT and Ifnar1 –/–mice 72 h post infection. E, F) HSP90α/β, Lcn2 and S100A9 were detected by immunoblot in the i.p. pIC model from cecum of WT (E) and Ifnar1 –/–(F) mice 72 h post infection from a representative experiment. Each dot represents one mouse, the geometric mean is indicated. P values were calculated by two-tailed Mann-Whitney test. *p value < 0.05, **p value < 0.01, ***p value < 0.001; ns, not significant. N of mice used in each group in (A, B, C, D): mock = 3 WT and 3 Ifnar1 –/–, pIC = 3–5 WT and 3–5 Ifnar1 –/–, pIC+STM = 7–10 WT and 7–10 Ifnar1 –/–, STM = 7–10 WT and 7–10 Ifnar1 –/–. N of mice used in each group in (E, F): mock = 1 WT and 1 Ifnar1 –/–, pIC = 4WT and 4 Ifnar1 –/–, pIC+STM = 5 WT and 5 Ifnar1 –/–, STM = 3 WT and 3 Ifnar1 –/–. The samples showed were pooled from two independent experiments in (A, B, C and D) or used from one representative experiment in (E, F). (TIF) [file ppat.1005572.s005.tif]

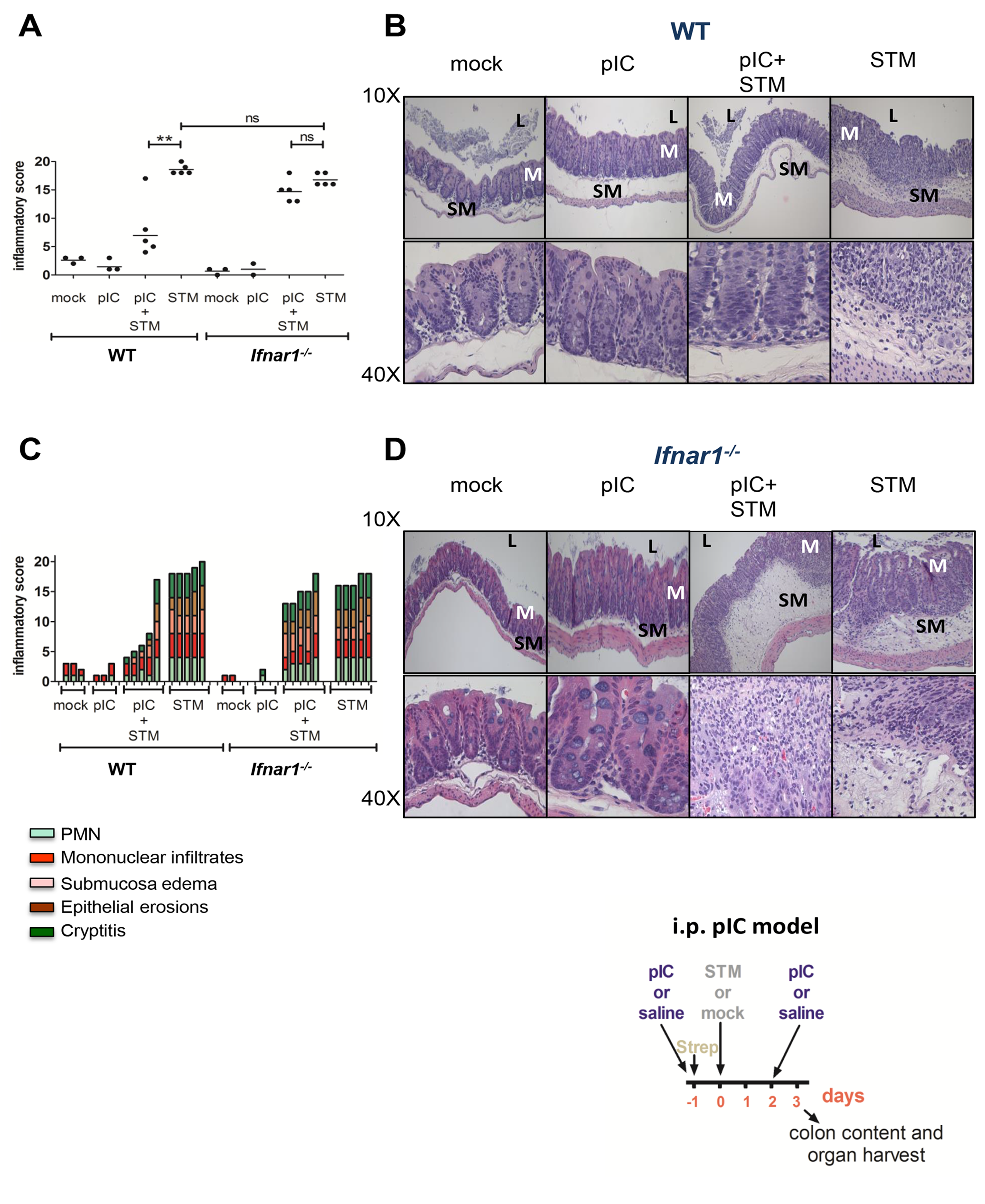

Supplement: S6 Fig — Blinded histopathology scores of cecal samples from WT (A, B and C) and Ifnar1 –/- (A, C and, D) mice at 72 h post S. Typhimurium or mock infection, i.p. pIC- or mock- treated. The score of individual mice (circles) and the geometric mean for each group (bars) are indicated in (A). P values were calculated by two-tailed Mann-Whitney test. **p < 0.01; ns, not significant. One representative experiment is shown. N of mice used in each group in (A, C): mock = 3 WT and 3 Ifnar1 –/-, pIC = 3 WT and 2 Ifnar1 –/-, pIC+STM = 5 WT and 5 Ifnar1 –/-, STM = 5 WT and 5 Ifnar1 –/-. A detailed scoring for the animals shown in (A) is provided; each stacked column represents an individual mouse in (C). B and D) Hematoxylin and eosin (H&E)-stained sections from representative animals for each group in WT (B) and Ifnar1 -/- (D) mice. Abbreviations are as follows: L, lumen; M, mucosa; SM, submucosa. (TIF) [file ppat.1005572.s006.tif]

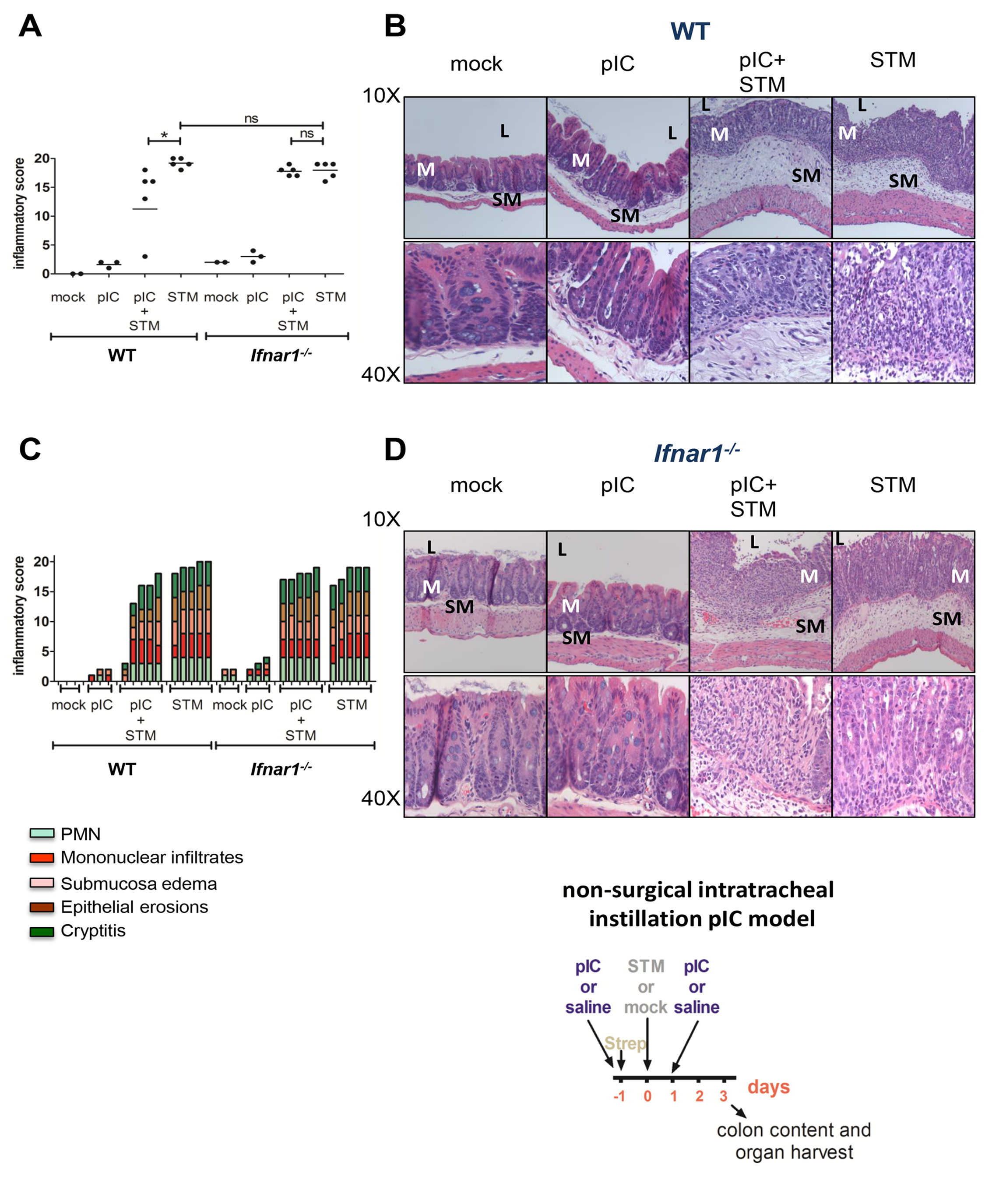

Supplement: S7 Fig — Blinded histopathology scores of cecal samples from WT (A, B and C) and Ifnar1 –/- (A, C and D) mice at 72 h post S. Typhimurium or mock infection, treated or not with pIC through non-surgical intratracheal instillation. The score of individual mice (circles) and the geometric mean for each group (bars) are indicated in (A). P values were calculated by two-tailed Mann-Whitney test. *p < 0.05; ns, not significant. One representative experiment is shown. N of mice used in each group in (A, C): mock = 2 WT and 2 Ifnar1 –/-, pIC = 3 WT and 3 Ifnar1 –/-, pIC+STM = 5 WT and 5 Ifnar1 –/-, STM = 5 WT and 5 Ifnar1 –/-. A detailed scoring for the animals shown in (A) is provided; each stacked column represents an individual mouse in (C). B and D) Hematoxylin and eosin (H&E)-stained sections from representative animals for each group in WT (B) and Ifnar1 -/- (D) mice. Abbreviations are as follows: L, lumen; M, mucosa; SM, submucosa. (TIF) [file ppat.1005572.s007.tif]
