# Supplementary material for: Influenza Virus Affects Intestinal Microbiota and Secondary Salmonella Infection in the Gut through Type I Interferons
Source: PLoS Pathog. 2016 May 5;12(5):e1005572. doi: 10.1371/journal.ppat.1005572 (PMC4858270; doi:10.1371/journal.ppat.1005572)
Supplement: S1 Table — (DOCX) [file ppat.1005572.s008.docx]

| **BACTERIAL STRAIN**  **(*S.* Typhimurium)** |  |  |
| --- | --- | --- |
| \| **Designation** \|  \| \| --- \| --- \| | **Genotype** | **Source or Reference** |
| IR715 | ATCC 14028, NalR derivative | [64] |
| **PLASMIDS** |  |  |
| pHP45omega | StrepR, CarbR | [65] |
| pSW195 | pCR2.1::*Salmonella* 16S rDNA | [18] |
| pSW196 | pCR2.1::*Enterobacteriaceae* 16S rDNA | [18] |
| pSW191 | pCR2.1::*Eubacteria* 16S rDNA | [18] |
| *SFB* plasmid | pCR2.1::*Segmented Filamentous Bacteria* 16S rDNA | [69] |
