# Supplementary material for: Influenza Virus Affects Intestinal Microbiota and Secondary Salmonella Infection in the Gut through Type I Interferons
Source: PLoS Pathog. 2016 May 5;12(5):e1005572. doi: 10.1371/journal.ppat.1005572 (PMC4858270; doi:10.1371/journal.ppat.1005572)
Supplement: S2 Table — (DOCX) [file ppat.1005572.s009.docx]

| **PRIMERS** |  |  |
| --- | --- | --- |
| **Species** | **Target** | **Primer Pairs** |
| *Mus musculus* | *L32* | 5’-TTAAGCGAAACTGGCGGAAAC-3  5’-TTGTTGCTCCCATAACCGATG-3' |
| *Mus musculus* | *Ifnγ* | 5’-TCAAGTGGCATAGATGTGGAAGAA-3’  5’-TGGCTCTGCAGGATTTTCATG-3’ |
| *Mus musculus* | *Lcn2* | 5’-ACATTTGTTCCAAGCTCCAGGGC-3  5’-CATGGCGAACTGGTTGTAGTCCG-3’ |
| *Mus musculus* | *S100A9* | 5’-GGTGGAAGCACAGTTGGCA-3’  5’-GTGTCCAGGTCCTCCATGATG-3’ |
| *Mus musculus* | *Cxcl2* | 5’-CCAACCACCAGGCTACAGG-3  5’-GCGTCACACTCAAGCTCTG-3’ |
| *Mus musculus* | *Il6* | 5’-ACAACCACGGCCTTCCCTACTT-3’  5’-CACGATTTCCCAGAGAACATGTG-3’ |
| *Mus musculus* | *Muc2* | 5’-CCCAGAAGGGACTGTGTATG-3’  5’-TTGTGTTCGCTCTTGGTCAG-3’ |
| *Mus musculus* | *Il10* | 5’-TCATCGATTTCTCCCCTGTGA-3’  5’-GACACCTTGGTCTTGGAGCTTATT-3’ |
| *Mus musculus* | *Cxcl10* | 5’-CCAGTGAGAATGAGGGCCATA-3’  5’-TCGTGGCAATGATCTCAACAC-3’ |
| *Mus musculus* | *Mx1* | 5’-AAACCTGATCCGACTTCACTTCC-3’  5’-TGATCGTCTTCAAGGTTTCCTTGT-3’ |
| PR8 | *M* | 5’-CATGGAATGGCTAAAGACAAGACC-3’  5’-CCATTAAGGGCATTTTGGACA-3’  [70] |
| *Eubacteria* | *16S rDNA* | Bac1  5’-CGCCCGCCGCGCCCCGCGCCCGTCCCGCCGCCCCCGCCCGACTACGTGCCAGCAGCC-3’  Bac2  5’-GGACTACCAGGGTATCTAATCC-3’  [68] |
| *Salmonella* | *16S rDNA* | Sal454 5’-TGTTGTGGTTAATAACCGCA-3’  Uni785R 5’-GACTACCAGGGTATCTAATCC-3’  [18] |
| *Enterobacteriaceae* | *16S rDNA* | Uni515F 5’-GTGCCAGCMGCCGGCGGTAA-3’  Ent826R 5’-GCCTCAAGGGCACAACCTCCAAG-3’  [18] |
| *Eubacteria* | *16S rDNA* | UniF340 5’-ACTCCTACGGGAGGCAGCAGT-3’  UniR514 5’-ATTACCGCGGCTGCTGGC-3’  [71] |
| *Segmented Filamentous Bacteria (SFB)* | *16S rDNA* | SFBF 5’-GACGCTGAGGCATGAGAGCAT-3’  SFBR 5’-GACGGCACGGATTGTTATTCA-3’  [69] |
