# Supplementary material for: Influenza Virus Affects Intestinal Microbiota and Secondary Salmonella Infection in the Gut through Type I Interferons
Source: PLoS Pathog. 2016 May 5;12(5):e1005572. doi: 10.1371/journal.ppat.1005572 (PMC4858270; doi:10.1371/journal.ppat.1005572)
Supplement: S1 References — (DOCX) [file ppat.1005572.s010.docx]

**S1 References**

69. Salzman NH, Hung K, Haribhai D, Chu H, Karlsson-Sjoberg J, Amir E, et al. Enteric defensins are essential regulators of intestinal microbial ecology. Nat Immunol. 2010;11(1):76-83.

70. Rosenberger CM, Podyminogin RL, Askovich PS, Navarro G, Kaiser SM, Sanders CJ, et al. Characterization of innate responses to influenza virus infection in a novel lung type I epithelial cell model. J Gen Virol. 2014;95(Pt 2):350-62.

71. Barman M, Unold D, Shifley K, Amir E, Hung K, Bos N, et al. Enteric salmonellosis disrupts the microbial ecology of the murine gastrointestinal tract. Infect Immun. 2008;76(3):907-15.
